# Supplementary material for: Sex-specific risks for cardiovascular disease across the glycaemic spectrum: a population-based cohort study using the UK Biobank
Source: Lancet Reg Health Eur. 2023 Aug 10;32:100693. doi: 10.1016/j.lanepe.2023.100693 (PMC10477037; doi:10.1016/j.lanepe.2023.100693)
Supplement: Supplementary Appendix [file mmc1.pdf]

Supplementary Appendix

Manuscript: Sex-specific risks for cardiovascular disease across the glycaemic spectrum: a population-based cohort study using the UK Biobank

Table of Contents

eTable 1. Codelists for outcomes, exposure, and covariates 2

eTable 2. Sex-specific associations between HbA1c category and cardiovascular diseases 4

eTable 3. Sex-specific associations amending the CAD outcome to include angina and restricting the stroke outcome to ischemic stroke only 6

eTable 4. Sensitivity analysis excluding individuals with HbA1c <20 mmol/mol 7

eTable 5. Sensitivity analysis excluding events occurring within 180 days of follow-up 9

eTable 6. Sensitivity analysis excluding individuals with diagnosed diabetes 11

eTable 7. Sensitivity analysis excluding individuals with any cardiovascular disease at baseline instead of outcome-specific exclusion 13

eTable 8. Sensitivity analysis using age as time scale in the Cox regression models 15

eTable 9. Sensitivity analysis including sex-confounder interactions in the Cox regression model 16

eFigure 1. Schoenfeld residuals testing the proportional hazards assumption between HbA1c category and any cardiovascular disease, by sex 17

The RECORD statement – checklist of items, extended from the STROBE statement, that should be reported in observational studies using routinely collected health data 18

**eTable 1.** Codelists for outcomes, exposure, and covariates		
**Outcomes (ICD-10 coded)**	**Primary analysis**	**Sensitivity analysis**
Coronary artery disease (CAD)	I21.*, I46.*	I21.*, I46.*, I20.*
Atrial fibrillation (Afib)	I48.*	-
Deep vein thrombosis (DVT)	I80.1, I80.2, I80.3	-
Pulmonary embolism (PE)	I26.*	-
Stroke	I60.*, I61.*, I62.*, I63.*, I64	I63.*
Heart failure (HF)	I50.*	-
**Exposure and covariates**	**Description**	**UKB Data-Field Number**
HbA1c	Blood biochemistry measured by HPLC analysis on a Bio-Rad VARIANT II Turbo	n_30750_*
Diagnosed diabetes	Validated algorithm from: Eastwood et al 2016, DOI: 10.1371/journal.pone.0162388	
Index of multiple deprivation	Townsend deprivation index calculated immediately prior to participant joining UK Biobank. Based on the preceding national census output areas. Each participant is assigned a score corresponding to the output area in which their postcode is located.	n_22189_*
Smoking status	ACE touchscreen question "Do you smoke tobacco now?"	n_1239_*
	ACE touchscreen question "How old were you when you first started smoking on most days?"	n_2867_*
Alcohol consumption	ACE touchscreen question "About how often do you drink alcohol?"	n_1558_*
Physical activity	ACE touchscreen question "In a typical WEEK, on how many days did you do 10 minutes or more of moderate physical activities like carrying light loads, cycling at normal pace? (Do not include walking)"	n_884_*
	ACE touchscreen question "In a typical WEEK, how many days did you do 10 minutes or more of vigorous physical activity? (These are activities that make you sweat or breathe hard such as fast cycling, aerobics, heavy lifting)"	n_904_*
Body mass index	Constructed from height and weight measured during the initial Assessment Centre visit	n_21001_*
Waist-hip ratio	Waist circumference measured during the initial Assessment Centre visit	n_48_*
	Hip circumference measured during the initial Assessment Centre visit	n_49_*
Processed meat consumption	ACE touchscreen question "How often do you eat processed meats (such as bacon, ham, sausages, meat pies, kebabs, burgers, chicken nuggets)?"	n_1349_*
Fruit/vegetable consumption	ACE touchscreen question "On average how many heaped tablespoons of COOKED vegetables would you eat per DAY? (Do not include potatoes; put '0' if you do not eat any)"	n_1289_*
	ACE touchscreen question "On average how many heaped tablespoons of SALAD or RAW vegetables would you eat per DAY? (Include lettuce, tomato in sandwiches; put '0' if you do not eat any)"	n_1299_*
	ACE touchscreen question "About how many pieces of FRESH fruit would you eat per DAY? (Count one apple, one banana, 10 grapes etc as one piece; put '0' if you do not eat any)"	n_1309_*
	ACE touchscreen question "About how many pieces of DRIED fruit would you eat per DAY? (Count one prune, one dried apricot, 10 raisins as one piece; put '0' if you do not eat any)"	n_1319_*
Total cholesterol	Blood biochemistry measured by CHO-POD analysis on a Beckman Coulter AU5800	n_30690_*
eGFR	Blood biochemistry measured by enzymatic analysis on a Beckman Coulter AU5800	n_30700_*
C-reactive protein	Blood biochemistry measured by immunoturbidimetric - high sensitivity analysis on a Beckman Coulter AU5800	n_30710_*
Hypertension	ACE touchscreen question "Has a doctor ever told you that you have had any of the following conditions? (You can select more than one answer)"	n_6150_*

	Coded by trained nurse during intake interview	n_20002_*
Antihypertensive medication	Coded by trained nurse during intake interview	n_20003_*
Statin	Coded by trained nurse during intake interview	n_20003_*
Family history of cardiovascular disease	ACE touchscreen question "Has/did your mother ever suffer from? (You can select more than one answer)"	n_20110_*
	ACE touchscreen question "Has/did your father ever suffer from? (You can select more than one answer)"	n_20107_*
	ACE touchscreen question "Have any of your brothers or sisters suffered from the any of the following diseases? (You can select more than one answer)"	n_20111_*
NB. For further details, please visit the UK Biobank Data Showcase at https://biobank.ndph.ox.ac.uk/showcase/index.cgi. The UKB Data-Field numbers above can be searched in the Data Showcase to access highly detailed notes about instruments used, validation exercises (where applicable), screenshots of the survey, coding of the variable, and summary statistics of each variable. Programming code we developed to clean and manipulate the raw UK Biobank data into the variables used in the present analysis can be found at the following dedicated GitHub repository: https://github.com/ehr-lshtm/ukb-a1c-cvd		

eTable 2. Sex-specific associations between HbA1c category and cardiovascular diseases

	Unadjusted		Age-adjusted		+ Socio-demographics		+ Lifestyle characteristics		+ Clinical characteristics	
	**HR**	**(95% CI)**	**HR**	**(95% CI)**	**HR**	**(95% CI)**	**HR**	**(95% CI)**	**HR**	**(95% CI)**
**Any cardiovascular disease***										
Men										
Low-normal HbA1c	0.72	(0.70-0.74)	0.86	(0.84-0.88)	0.87	(0.84-0.89)	0.95	(0.92-0.97)	0.98	(0.96-1.01)
Normal HbA1c	1.00	ref	1.00	ref	1.00	ref	1.00	ref	1.00	ref
Pre-diabetes	1.46	(1.38-1.54)	1.30	(1.24-1.38)	1.29	(1.22-1.36)	1.09	(1.03-1.15)	1.04	(0.98-1.10)
Undiagnosed diabetes	1.32	(1.19-1.46)	1.31	(1.18-1.45)	1.28	(1.16-1.42)	1.00	(0.90-1.11)	0.97	(0.88-1.08)
Diagnosed diabetes	1.82	(1.75-1.89)	1.55	(1.49-1.61)	1.53	(1.47-1.59)	1.26	(1.21-1.32)	1.06	(1.02-1.11)
Women										
Low-normal HbA1c	0.64	(0.62-0.65)	0.86	(0.84-0.89)	0.87	(0.84-0.89)	0.96	(0.93-0.99)	0.99	(0.96-1.02)
Normal HbA1c	1.00	ref	1.00	ref	1.00	ref	1.00	ref	1.00	ref
Pre-diabetes	1.67	(1.57-1.77)	1.47	(1.38-1.56)	1.46	(1.38-1.55)	1.18	(1.11-1.26)	1.11	(1.04-1.18)
Undiagnosed diabetes	1.38	(1.18-1.62)	1.33	(1.14-1.56)	1.32	(1.12-1.54)	0.93	(0.79-1.09)	0.88	(0.75-1.03)
Diagnosed diabetes	2.19	(2.07-2.32)	2.00	(1.89-2.12)	1.95	(1.84-2.07)	1.40	(1.32-1.48)	1.17	(1.10-1.24)
p-interaction		*<0.0001*		*<0.0001*		*<0.0001*		*0.0197*		*0.0387*
**Coronary artery disease**										
Men										
Low-normal HbA1c	0.64	(0.61-0.68)	0.74	(0.71-0.78)	0.75	(0.71-0.79)	0.85	(0.80-0.89)	0.90	(0.86-0.95)
Normal HbA1c	1.00	ref	1.00	ref	1.00	ref	1.00	ref	1.00	ref
Pre-diabetes	1.58	(1.43-1.75)	1.44	(1.30-1.59)	1.41	(1.28-1.56)	1.21	(1.09-1.34)	1.15	(1.04-1.27)
Undiagnosed diabetes	1.76	(1.48-2.10)	1.74	(1.46-2.07)	1.69	(1.42-2.01)	1.38	(1.16-1.65)	1.33	(1.11-1.59)
Diagnosed diabetes	2.06	(1.92-2.21)	1.82	(1.69-1.95)	1.76	(1.64-1.89)	1.53	(1.42-1.65)	1.51	(1.39-1.63)
Women										
Low-normal HbA1c	0.56	(0.52-0.60)	0.71	(0.65-0.76)	0.71	(0.66-0.77)	0.82	(0.76-0.89)	0.89	(0.82-0.96)
Normal HbA1c	1.00	ref	1.00	ref	1.00	ref	1.00	ref	1.00	ref
Pre-diabetes	1.70	(1.46-1.96)	1.53	(1.32-1.77)	1.52	(1.31-1.76)	1.19	(1.03-1.38)	1.12	(0.97-1.30)
Undiagnosed diabetes	1.57	(1.09-2.25)	1.52	(1.06-2.18)	1.48	(1.03-2.12)	1.05	(0.73-1.50)	0.99	(0.69-1.42)
Diagnosed diabetes	2.76	(2.44-3.12)	2.55	(2.26-2.88)	2.46	(2.18-2.79)	1.79	(1.57-2.03)	1.80	(1.58-2.05)
p-interaction		*<0.0001*		*<0.0001*		*<0.0001*		*0.1037*		*0.0540*
**Atrial fibrillation**										
Men										
Low-normal HbA1c	0.75	(0.72-0.77)	0.94	(0.91-0.98)	0.94	(0.91-0.98)	1.02	(0.98-1.06)	1.04	(1.01-1.08)
Normal HbA1c	1.00	ref	1.00	ref	1.00	ref	1.00	ref	1.00	ref
Pre-diabetes	1.50	(1.40-1.62)	1.30	(1.20-1.40)	1.31	(1.21-1.41)	1.09	(1.01-1.18)	1.03	(0.96-1.11)
Undiagnosed diabetes	1.37	(1.18-1.58)	1.35	(1.17-1.56)	1.36	(1.18-1.57)	1.02	(0.88-1.18)	1.01	(0.87-1.17)
Diagnosed diabetes	1.92	(1.82-2.02)	1.58	(1.50-1.67)	1.58	(1.50-1.67)	1.25	(1.19-1.33)	1.02	(0.96-1.08)
Women										
Low-normal HbA1c	0.67	(0.64-0.70)	0.98	(0.94-1.03)	0.98	(0.94-1.03)	1.06	(1.01-1.11)	1.06	(1.01-1.11)
Normal HbA1c	1.00	ref	1.00	ref	1.00	ref	1.00	ref	1.00	ref
Pre-diabetes	1.59	(1.45-1.74)	1.35	(1.23-1.48)	1.37	(1.24-1.50)	1.12	(1.02-1.24)	1.04	(0.95-1.15)
Undiagnosed diabetes	1.29	(1.01-1.65)	1.22	(0.96-1.56)	1.25	(0.98-1.60)	0.90	(0.70-1.15)	0.85	(0.66-1.09)
Diagnosed diabetes	2.16	(1.98-2.35)	1.90	(1.74-2.07)	1.90	(1.75-2.07)	1.35	(1.24-1.47)	1.06	(0.97-1.16)
p-interaction		*<0.0001*		*0.0081*		*0.0092*		*0.3822*		*0.6340*
**Deep vein thrombosis**										
Men										
Low-normal HbA1c	0.85	(0.77-0.93)	0.97	(0.88-1.06)	0.97	(0.88-1.06)	1.07	(0.97-1.18)	1.07	(0.97-1.17)
Normal HbA1c	1.00	ref	1.00	ref	1.00	ref	1.00	ref	1.00	ref
Pre-diabetes	1.22	(0.98-1.51)	1.11	(0.90-1.38)	1.10	(0.89-1.37)	0.92	(0.74-1.14)	0.92	(0.74-1.14)
Undiagnosed diabetes	1.14	(0.76-1.73)	1.13	(0.75-1.71)	1.12	(0.74-1.69)	0.84	(0.55-1.27)	0.84	(0.56-1.27)
Diagnosed diabetes	1.47	(1.26-1.72)	1.31	(1.12-1.53)	1.30	(1.11-1.52)	1.04	(0.88-1.21)	1.09	(0.92-1.28)
Women										
Low-normal HbA1c	0.67	(0.60-0.74)	0.83	(0.75-0.92)	0.84	(0.75-0.93)	0.95	(0.85-1.06)	0.94	(0.85-1.05)
Normal HbA1c	1.00	ref	1.00	ref	1.00	ref	1.00	ref	1.00	ref
Pre-diabetes	1.47	(1.18-1.83)	1.34	(1.08-1.66)	1.33	(1.07-1.65)	1.03	(0.83-1.29)	1.02	(0.82-1.27)
Undiagnosed diabetes	1.52	(0.91-2.52)	1.47	(0.88-2.46)	1.46	(0.88-2.44)	0.98	(0.59-1.64)	0.98	(0.58-1.63)
Diagnosed diabetes	1.61	(1.30-2.00)	1.50	(1.21-1.86)	1.47	(1.19-1.82)	0.99	(0.79-1.23)	1.02	(0.82-1.28)
p-interaction		*0.0013*		*0.0408*		*0.0451*		*0.3596*		*0.3659*
**Pulmonary embolism**										
Men										
Low-normal HbA1c	0.76	(0.70-0.82)	0.88	(0.81-0.95)	0.88	(0.81-0.95)	0.98	(0.91-1.06)	0.99	(0.92-1.07)
Normal HbA1c	1.00	ref	1.00	ref	1.00	ref	1.00	ref	1.00	ref
Pre-diabetes	1.47	(1.25-1.72)	1.34	(1.14-1.57)	1.33	(1.13-1.56)	1.07	(0.91-1.26)	1.07	(0.91-1.25)
Undiagnosed diabetes	1.33	(0.98-1.81)	1.32	(0.97-1.79)	1.30	(0.96-1.77)	0.94	(0.69-1.28)	0.93	(0.68-1.26)
Diagnosed diabetes	1.37	(1.21-1.56)	1.21	(1.07-1.38)	1.20	(1.05-1.36)	0.92	(0.81-1.05)	0.99	(0.86-1.14)
Women										
Low-normal HbA1c	0.61	(0.56-0.66)	0.78	(0.72-0.84)	0.78	(0.72-0.84)	0.89	(0.82-0.97)	0.90	(0.83-0.98)
Normal HbA1c	1.00	ref	1.00	ref	1.00	ref	1.00	ref	1.00	ref

Pre-diabetes	1.59	(1.36-1.87)	1.44	(1.23-1.69)	1.43	(1.22-1.68)	1.09	(0.93-1.28)	1.08	(0.91-1.27)
Undiagnosed diabetes	1.17	(0.75-1.81)	1.13	(0.73-1.75)	1.12	(0.72-1.74)	0.73	(0.47-1.14)	0.72	(0.46-1.12)
Diagnosed diabetes	1.64	(1.39-1.93)	1.51	(1.29-1.78)	1.48	(1.26-1.75)	0.98	(0.83-1.16)	1.03	(0.87-1.23)
p-interaction	*0.0001*		*0.0125*		*0.0151*		*0.3246*		*0.3673*	
**Stroke**										
Men										
Low-normal HbA1c	0.71	(0.67-0.75)	0.87	(0.82-0.92)	0.88	(0.82-0.93)	0.94	(0.89-1.00)	0.97	(0.92-1.04)
Normal HbA1c	1.00	ref	1.00	ref	1.00	ref	1.00	ref	1.00	ref
Pre-diabetes	1.44	(1.27-1.64)	1.26	(1.11-1.43)	1.23	(1.08-1.40)	1.10	(0.96-1.25)	1.04	(0.92-1.19)
Undiagnosed diabetes	1.52	(1.21-1.91)	1.50	(1.19-1.88)	1.45	(1.15-1.82)	1.23	(0.98-1.55)	1.19	(0.95-1.51)
Diagnosed diabetes	2.15	(1.97-2.34)	1.81	(1.66-1.97)	1.76	(1.61-1.91)	1.59	(1.46-1.74)	1.43	(1.30-1.57)
Women										
Low-normal HbA1c	0.65	(0.61-0.70)	0.91	(0.85-0.98)	0.92	(0.85-0.99)	0.98	(0.91-1.06)	1.01	(0.94-1.08)
Normal HbA1c	1.00	ref	1.00	ref	1.00	ref	1.00	ref	1.00	ref
Pre-diabetes	1.65	(1.44-1.91)	1.42	(1.24-1.64)	1.39	(1.20-1.60)	1.22	(1.06-1.41)	1.14	(0.99-1.32)
Undiagnosed diabetes	1.33	(0.91-1.93)	1.26	(0.87-1.84)	1.22	(0.84-1.77)	1.01	(0.70-1.47)	0.95	(0.65-1.39)
Diagnosed diabetes	2.28	(2.01-2.59)	2.02	(1.78-2.30)	1.94	(1.71-2.20)	1.63	(1.43-1.86)	1.43	(1.25-1.64)
p-interaction	*0.1116*		*0.3654*		*0.4287*		*0.6118*		*0.6596*	
**Heart failure**										
Men										
Low-normal HbA1c	0.62	(0.59-0.66)	0.78	(0.74-0.83)	0.79	(0.75-0.84)	0.93	(0.88-0.98)	1.00	(0.94-1.05)
Normal HbA1c	1.00	ref	1.00	ref	1.00	ref	1.00	ref	1.00	ref
Pre-diabetes	1.91	(1.73-2.10)	1.65	(1.50-1.82)	1.60	(1.45-1.76)	1.20	(1.09-1.33)	1.08	(0.98-1.19)
Undiagnosed diabetes	1.99	(1.67-2.36)	1.96	(1.65-2.33)	1.86	(1.56-2.21)	1.21	(1.01-1.44)	1.15	(0.96-1.37)
Diagnosed diabetes	3.02	(2.83-3.22)	2.51	(2.35-2.68)	2.39	(2.24-2.56)	1.69	(1.58-1.81)	1.27	(1.18-1.36)
Women										
Low-normal HbA1c	0.61	(0.56-0.65)	0.88	(0.82-0.94)	0.89	(0.83-0.95)	1.04	(0.97-1.12)	1.09	(1.01-1.17)
Normal HbA1c	1.00	ref	1.00	ref	1.00	ref	1.00	ref	1.00	ref
Pre-diabetes	2.43	(2.16-2.73)	2.07	(1.84-2.32)	2.00	(1.78-2.25)	1.43	(1.27-1.61)	1.25	(1.11-1.41)
Undiagnosed diabetes	2.27	(1.71-3.01)	2.16	(1.62-2.86)	2.05	(1.54-2.73)	1.19	(0.89-1.58)	1.06	(0.79-1.40)
Diagnosed diabetes	4.19	(3.80-4.61)	3.69	(3.35-4.07)	3.46	(3.14-3.82)	2.00	(1.80-2.21)	1.44	(1.30-1.60)
p-interaction	*<0.0001*		*<0.0001*		*<0.0001*		*0.0107*		*0.0678*	

*A composite measure of all examined outcomes

Abbreviations: HbA1c, glycated haemoglobin; HR, hazard ratio; CI, confidence interval

Notes: Categories were defined by baseline HbA1c levels as follows: low-normal (<35 mmol/mol or <5.5%), normal (35-41 mmol/mol or 5.5-5.9%), pre-diabetes (42-47 mmol/mol or 6.0-6.4%), undiagnosed diabetes (≥48 mmol/mol or ≥6.5%), or diagnosed diabetes. Sex-specific hazard ratios from Cox proportional hazards models, adjusted for age at study entry, socio-demographics (i.e., ethnicity, deprivation), lifestyle characteristics (i.e., smoking status, alcohol consumption, physical activity, body mass index, waist-hip ratio, processed meat and fruit and vegetable intake), and clinical characteristics (i.e., total cholesterol, estimated glomerular filtration rate, C-reactive protein, diagnosed hypertension, use of antihypertensive medication or statins, and family history of cardiovascular disease).

eTable 3. Sex-specific associations amending the CAD outcome to include angina and restricting the stroke outcome to ischemic stroke only

	**Unadjusted**		**Age-adjusted**		**+ Socio-demographics**		**+ Lifestyle characteristics**		**+ Clinical characteristics**	
	**HR**	**(95% CI)**	**HR**	**(95% CI)**	**HR**	**(95% CI)**	**HR**	**(95% CI)**	**HR**	**(95% CI)**
**Coronary artery disease (primary analysis)**										
Men										
Low-normal HbA1c	0.64	(0.61-0.68)	0.74	(0.71-0.78)	0.75	(0.71-0.79)	0.85	(0.80-0.89)	0.90	(0.86-0.95)
Normal HbA1c	1.00	ref	1.00	ref	1.00	ref	1.00	ref	1.00	ref
Pre-diabetes	1.58	(1.43-1.75)	1.44	(1.30-1.59)	1.41	(1.28-1.56)	1.21	(1.09-1.34)	1.15	(1.04-1.27)
Undiagnosed diabetes	1.76	(1.48-2.10)	1.74	(1.46-2.07)	1.69	(1.42-2.01)	1.38	(1.16-1.65)	1.33	(1.11-1.59)
Diagnosed diabetes	2.06	(1.92-2.21)	1.82	(1.69-1.95)	1.76	(1.64-1.89)	1.53	(1.42-1.65)	1.51	(1.39-1.63)
Women										
Low-normal HbA1c	0.56	(0.52-0.60)	0.71	(0.65-0.76)	0.71	(0.66-0.77)	0.82	(0.76-0.89)	0.89	(0.82-0.96)
Normal HbA1c	1.00	ref	1.00	ref	1.00	ref	1.00	ref	1.00	ref
Pre-diabetes	1.70	(1.46-1.96)	1.53	(1.32-1.77)	1.52	(1.31-1.76)	1.19	(1.03-1.38)	1.12	(0.97-1.30)
Undiagnosed diabetes	1.57	(1.09-2.25)	1.52	(1.06-2.18)	1.48	(1.03-2.12)	1.05	(0.73-1.50)	0.99	(0.69-1.42)
Diagnosed diabetes	2.76	(2.44-3.12)	2.55	(2.26-2.88)	2.46	(2.18-2.79)	1.79	(1.57-2.03)	1.80	(1.58-2.05)
p-interaction	*<0.0001*		*<0.0001*		*<0.0001*		*0.1037*		*0.0540*	
**Coronary artery disease including angina (sensitivity analysis)**										
Men										
Low-normal HbA1c	0.65	(0.62-0.67)	0.76	(0.73-0.78)	0.76	(0.74-0.79)	0.85	(0.81-0.88)	0.91	(0.88-0.95)
Normal HbA1c	1.00	ref	1.00	ref	1.00	ref	1.00	ref	1.00	ref
Pre-diabetes	1.55	(1.44-1.68)	1.41	(1.31-1.52)	1.38	(1.28-1.49)	1.17	(1.08-1.26)	1.09	(1.01-1.17)
Undiagnosed diabetes	1.42	(1.23-1.64)	1.41	(1.22-1.63)	1.36	(1.18-1.57)	1.09	(0.94-1.26)	1.06	(0.91-1.22)
Diagnosed diabetes	2.10	(1.99-2.21)	1.83	(1.74-1.93)	1.77	(1.68-1.87)	1.48	(1.40-1.57)	1.17	(1.11-1.24)
Women										
Low-normal HbA1c	0.56	(0.53-0.59)	0.73	(0.69-0.77)	0.73	(0.70-0.77)	0.84	(0.80-0.88)	0.90	(0.86-0.95)
Normal HbA1c	1.00	ref	1.00	ref	1.00	ref	1.00	ref	1.00	ref
Pre-diabetes	1.82	(1.66-2.00)	1.64	(1.49-1.80)	1.61	(1.47-1.77)	1.27	(1.16-1.39)	1.16	(1.06-1.27)
Undiagnosed diabetes	1.70	(1.36-2.13)	1.64	(1.31-2.06)	1.58	(1.26-1.98)	1.08	(0.86-1.36)	1.00	(0.80-1.26)
Diagnosed diabetes	2.73	(2.52-2.96)	2.52	(2.32-2.73)	2.42	(2.23-2.62)	1.69	(1.55-1.84)	1.34	(1.23-1.46)
p-interaction	*<0.0001*		*<0.0001*		*<0.0001*		*0.0671*		*0.0574*	
**Any stroke (primary analysis)**										
Men										
Low-normal HbA1c	0.71	(0.67-0.75)	0.87	(0.82-0.92)	0.88	(0.82-0.93)	0.94	(0.89-1.00)	0.97	(0.92-1.04)
Normal HbA1c	1.00	ref	1.00	ref	1.00	ref	1.00	ref	1.00	ref
Pre-diabetes	1.44	(1.27-1.64)	1.26	(1.11-1.43)	1.23	(1.08-1.40)	1.10	(0.96-1.25)	1.04	(0.92-1.19)
Undiagnosed diabetes	1.52	(1.21-1.91)	1.50	(1.19-1.88)	1.45	(1.15-1.82)	1.23	(0.98-1.55)	1.19	(0.95-1.51)
Diagnosed diabetes	2.15	(1.97-2.34)	1.81	(1.66-1.97)	1.76	(1.61-1.91)	1.59	(1.46-1.74)	1.43	(1.30-1.57)
Women										
Low-normal HbA1c	0.65	(0.61-0.70)	0.91	(0.85-0.98)	0.92	(0.85-0.99)	0.98	(0.91-1.06)	1.01	(0.94-1.08)
Normal HbA1c	1.00	ref	1.00	ref	1.00	ref	1.00	ref	1.00	ref
Pre-diabetes	1.65	(1.44-1.91)	1.42	(1.24-1.64)	1.39	(1.20-1.60)	1.22	(1.06-1.41)	1.14	(0.99-1.32)
Undiagnosed diabetes	1.33	(0.91-1.93)	1.26	(0.87-1.84)	1.22	(0.84-1.77)	1.01	(0.70-1.47)	0.95	(0.65-1.39)
Diagnosed diabetes	2.28	(2.01-2.59)	2.02	(1.78-2.30)	1.94	(1.71-2.20)	1.63	(1.43-1.86)	1.43	(1.25-1.64)
p-interaction	*0.1116*		*0.3654*		*0.4287*		*0.6118*		*0.6596*	
**Ischemic stroke (sensitivity analysis)**										
Men										
Low-normal HbA1c	0.68	(0.63-0.73)	0.83	(0.78-0.90)	0.84	(0.78-0.91)	0.92	(0.85-0.99)	0.96	(0.89-1.03)
Normal HbA1c	1.00	ref	1.00	ref	1.00	ref	1.00	ref	1.00	ref
Pre-diabetes	1.49	(1.28-1.73)	1.30	(1.12-1.51)	1.26	(1.09-1.47)	1.10	(0.95-1.28)	1.05	(0.90-1.22)
Undiagnosed diabetes	1.65	(1.27-2.15)	1.62	(1.25-2.11)	1.56	(1.20-2.03)	1.29	(0.99-1.68)	1.25	(0.96-1.62)
Diagnosed diabetes	2.29	(2.07-2.52)	1.91	(1.73-2.11)	1.85	(1.68-2.05)	1.64	(1.48-1.82)	1.50	(1.34-1.67)
Women										
Low-normal HbA1c	0.61	(0.56-0.67)	0.86	(0.78-0.94)	0.87	(0.79-0.95)	0.94	(0.86-1.04)	0.97	(0.89-1.07)
Normal HbA1c	1.00	ref	1.00	ref	1.00	ref	1.00	ref	1.00	ref
Pre-diabetes	1.68	(1.41-2.01)	1.44	(1.21-1.72)	1.40	(1.17-1.66)	1.20	(1.00-1.43)	1.11	(0.93-1.33)
Undiagnosed diabetes	1.87	(1.26-2.77)	1.77	(1.19-2.63)	1.69	(1.14-2.52)	1.35	(0.91-2.01)	1.26	(0.85-1.88)
Diagnosed diabetes	2.70	(2.33-3.13)	2.38	(2.05-2.76)	2.27	(1.95-2.63)	1.84	(1.58-2.15)	1.64	(1.40-1.92)
p-interaction	*0.0364*		*0.1971*		*0.2723*		*0.7787*		*0.8940*	

Abbreviations: HbA1c, glycated haemoglobin; HR, hazard ratio; CI, confidence interval

Notes: Categories were defined by baseline HbA1c levels as follows: low-normal (<35 mmol/mol or <5.5%), normal (35-41 mmol/mol or 5.5-5.9%), pre-diabetes (42-47 mmol/mol or 6.0-6.4%), undiagnosed diabetes (≥48 mmol/mol or ≥6.5%), or diagnosed diabetes. In primary analyses, coronary artery disease included myocardial infarction and sudden cardiac arrest. This table additionally includes angina in the outcome definition.

eTable 4. Sensitivity analysis excluding individuals with HbA1c <20 mmol/mol

	**Unadjusted**		**Age-adjusted**		**+ Socio-demographics**		**+ Lifestyle characteristics**		**+ Clinical characteristics**	
	**HR**	**(95% CI)**	**HR**	**(95% CI)**	**HR**	**(95% CI)**	**HR**	**(95% CI)**	**HR**	**(95% CI)**
**Any cardiovascular disease***										
Men										
Low-normal HbA1c	0.72	(0.70-0.74)	0.86	(0.84-0.88)	0.87	(0.84-0.89)	0.95	(0.92-0.97)	0.98	(0.96-1.01)
Normal HbA1c	1.00	ref	1.00	ref	1.00	ref	1.00	ref	1.00	ref
Pre-diabetes	1.46	(1.38-1.54)	1.30	(1.24-1.38)	1.29	(1.22-1.36)	1.09	(1.03-1.15)	1.04	(0.98-1.10)
Undiagnosed diabetes	1.32	(1.19-1.46)	1.31	(1.18-1.45)	1.28	(1.16-1.42)	1.00	(0.90-1.11)	0.97	(0.88-1.08)
Diagnosed diabetes	1.82	(1.75-1.89)	1.55	(1.49-1.61)	1.53	(1.47-1.59)	1.26	(1.21-1.31)	1.06	(1.02-1.11)
Women										
Low-normal HbA1c	0.64	(0.62-0.65)	0.86	(0.84-0.89)	0.87	(0.84-0.89)	0.96	(0.93-0.99)	0.98	(0.96-1.02)
Normal HbA1c	1.00	ref	1.00	ref	1.00	ref	1.00	ref	1.00	ref
Pre-diabetes	1.67	(1.57-1.77)	1.47	(1.38-1.56)	1.46	(1.38-1.55)	1.18	(1.11-1.26)	1.11	(1.04-1.18)
Undiagnosed diabetes	1.38	(1.18-1.62)	1.33	(1.14-1.56)	1.32	(1.12-1.54)	0.93	(0.79-1.09)	0.88	(0.75-1.03)
Diagnosed diabetes	2.19	(2.07-2.32)	2.00	(1.89-2.12)	1.95	(1.84-2.07)	1.40	(1.32-1.48)	1.17	(1.10-1.24)
p-interaction	*<0.0001*		*<0.0001*		*<0.0001*		*0.0197*		*0.0379*	
**Coronary artery disease**										
Men										
Low-normal HbA1c	0.64	(0.61-0.68)	0.75	(0.71-0.78)	0.75	(0.71-0.79)	0.85	(0.80-0.89)	0.90	(0.86-0.95)
Normal HbA1c	1.00	ref	1.00	ref	1.00	ref	1.00	ref	1.00	ref
Pre-diabetes	1.58	(1.43-1.75)	1.44	(1.30-1.59)	1.41	(1.28-1.56)	1.21	(1.09-1.34)	1.15	(1.04-1.27)
Undiagnosed diabetes	1.76	(1.48-2.10)	1.74	(1.46-2.07)	1.69	(1.42-2.01)	1.38	(1.16-1.65)	1.33	(1.11-1.59)
Diagnosed diabetes	2.06	(1.92-2.21)	1.82	(1.69-1.95)	1.76	(1.64-1.89)	1.53	(1.42-1.65)	1.51	(1.39-1.63)
Women										
Low-normal HbA1c	0.56	(0.52-0.60)	0.71	(0.65-0.77)	0.71	(0.66-0.77)	0.82	(0.76-0.89)	0.89	(0.82-0.96)
Normal HbA1c	1.00	ref	1.00	ref	1.00	ref	1.00	ref	1.00	ref
Pre-diabetes	1.70	(1.46-1.96)	1.53	(1.32-1.77)	1.52	(1.31-1.76)	1.19	(1.03-1.38)	1.12	(0.97-1.30)
Undiagnosed diabetes	1.57	(1.09-2.25)	1.52	(1.06-2.18)	1.48	(1.03-2.12)	1.05	(0.73-1.50)	0.99	(0.69-1.42)
Diagnosed diabetes	2.76	(2.44-3.12)	2.55	(2.26-2.88)	2.46	(2.18-2.79)	1.79	(1.57-2.03)	1.80	(1.58-2.05)
p-interaction	*<0.0001*		*<0.0001*		*<0.0001*		*0.1054*		*0.0548*	
**Atrial fibrillation**										
Men										
Low-normal HbA1c	0.75	(0.72-0.77)	0.94	(0.91-0.98)	0.94	(0.91-0.98)	1.02	(0.98-1.06)	1.04	(1.01-1.08)
Normal HbA1c	1.00	ref	1.00	ref	1.00	ref	1.00	ref	1.00	ref
Pre-diabetes	1.50	(1.40-1.62)	1.30	(1.20-1.40)	1.31	(1.21-1.41)	1.09	(1.01-1.18)	1.03	(0.96-1.11)
Undiagnosed diabetes	1.37	(1.18-1.58)	1.35	(1.17-1.56)	1.36	(1.18-1.57)	1.02	(0.88-1.18)	1.01	(0.87-1.17)
Diagnosed diabetes	1.92	(1.82-2.02)	1.58	(1.50-1.67)	1.58	(1.50-1.67)	1.25	(1.19-1.33)	1.02	(0.96-1.08)
Women										
Low-normal HbA1c	0.67	(0.64-0.70)	0.98	(0.94-1.03)	0.98	(0.94-1.03)	1.06	(1.01-1.11)	1.06	(1.01-1.11)
Normal HbA1c	1.00	ref	1.00	ref	1.00	ref	1.00	ref	1.00	ref
Pre-diabetes	1.59	(1.45-1.74)	1.35	(1.23-1.48)	1.37	(1.24-1.50)	1.12	(1.02-1.24)	1.04	(0.95-1.15)
Undiagnosed diabetes	1.29	(1.01-1.65)	1.22	(0.96-1.56)	1.25	(0.98-1.60)	0.90	(0.70-1.15)	0.85	(0.66-1.09)
Diagnosed diabetes	2.16	(1.98-2.35)	1.90	(1.74-2.07)	1.90	(1.75-2.07)	1.35	(1.24-1.47)	1.06	(0.97-1.16)
p-interaction	*<0.0001*		*0.0081*		*0.0092*		*0.3787*		*0.6329*	
**Deep vein thrombosis**										
Men										
Low-normal HbA1c	0.84	(0.77-0.92)	0.96	(0.88-1.06)	0.97	(0.88-1.06)	1.07	(0.97-1.17)	1.06	(0.97-1.17)
Normal HbA1c	1.00	ref	1.00	ref	1.00	ref	1.00	ref	1.00	ref
Pre-diabetes	1.22	(0.98-1.51)	1.11	(0.90-1.38)	1.10	(0.89-1.37)	0.92	(0.74-1.14)	0.92	(0.74-1.14)
Undiagnosed diabetes	1.14	(0.76-1.73)	1.13	(0.75-1.71)	1.12	(0.74-1.69)	0.84	(0.55-1.27)	0.84	(0.56-1.28)
Diagnosed diabetes	1.47	(1.26-1.72)	1.31	(1.12-1.53)	1.30	(1.12-1.52)	1.04	(0.88-1.22)	1.09	(0.92-1.28)
Women										
Low-normal HbA1c	0.67	(0.60-0.74)	0.83	(0.75-0.93)	0.84	(0.75-0.93)	0.95	(0.86-1.06)	0.95	(0.85-1.05)
Normal HbA1c	1.00	ref	1.00	ref	1.00	ref	1.00	ref	1.00	ref
Pre-diabetes	1.47	(1.18-1.83)	1.34	(1.08-1.67)	1.33	(1.07-1.65)	1.03	(0.83-1.29)	1.02	(0.82-1.28)
Undiagnosed diabetes	1.52	(0.91-2.52)	1.47	(0.88-2.46)	1.46	(0.88-2.44)	0.98	(0.59-1.64)	0.98	(0.58-1.63)
Diagnosed diabetes	1.61	(1.30-2.00)	1.50	(1.21-1.86)	1.47	(1.19-1.83)	0.99	(0.79-1.23)	1.02	(0.82-1.28)
p-interaction	*0.0017*		*0.0482*		*0.0532*		*0.3951*		*0.4011*	
**Pulmonary embolism**										
Men										
Low-normal HbA1c	0.76	(0.70-0.82)	0.88	(0.81-0.95)	0.88	(0.82-0.95)	0.98	(0.91-1.06)	0.99	(0.92-1.07)
Normal HbA1c	1.00	ref	1.00	ref	1.00	ref	1.00	ref	1.00	ref
Pre-diabetes	1.47	(1.25-1.72)	1.34	(1.14-1.57)	1.33	(1.13-1.56)	1.07	(0.91-1.26)	1.07	(0.91-1.25)
Undiagnosed diabetes	1.33	(0.98-1.81)	1.32	(0.97-1.79)	1.30	(0.96-1.77)	0.94	(0.69-1.28)	0.93	(0.68-1.26)
Diagnosed diabetes	1.37	(1.21-1.56)	1.21	(1.07-1.38)	1.20	(1.05-1.36)	0.92	(0.81-1.05)	0.99	(0.87-1.14)
Women										
Low-normal HbA1c	0.61	(0.56-0.66)	0.78	(0.71-0.84)	0.78	(0.72-0.84)	0.89	(0.82-0.97)	0.90	(0.83-0.98)
Normal HbA1c	1.00	ref	1.00	ref	1.00	ref	1.00	ref	1.00	ref

Pre-diabetes	1.59	(1.36-1.87)	1.44	(1.23-1.69)	1.43	(1.22-1.68)	1.09	(0.93-1.29)	1.08	(0.92-1.27)
Undiagnosed diabetes	1.17	(0.75-1.81)	1.13	(0.73-1.75)	1.12	(0.72-1.74)	0.73	(0.47-1.14)	0.72	(0.46-1.12)
Diagnosed diabetes	1.64	(1.39-1.93)	1.51	(1.29-1.78)	1.48	(1.26-1.75)	0.98	(0.83-1.16)	1.03	(0.87-1.23)
p-interaction	*0.0001*		*0.0115*		*0.0139*		*0.3094*		*0.3507*	
**Stroke**										
Men										
Low-normal HbA1c	0.71	(0.67-0.75)	0.87	(0.82-0.92)	0.88	(0.82-0.93)	0.94	(0.89-1.00)	0.98	(0.92-1.04)
Normal HbA1c	1.00	ref	1.00	ref	1.00	ref	1.00	ref	1.00	ref
Pre-diabetes	1.44	(1.27-1.64)	1.26	(1.11-1.43)	1.23	(1.08-1.40)	1.10	(0.96-1.25)	1.04	(0.92-1.19)
Undiagnosed diabetes	1.52	(1.21-1.91)	1.50	(1.19-1.88)	1.45	(1.15-1.82)	1.23	(0.98-1.55)	1.20	(0.95-1.51)
Diagnosed diabetes	2.15	(1.97-2.34)	1.81	(1.66-1.97)	1.76	(1.61-1.92)	1.59	(1.46-1.74)	1.43	(1.30-1.57)
Women										
Low-normal HbA1c	0.65	(0.61-0.70)	0.91	(0.84-0.98)	0.92	(0.85-0.98)	0.98	(0.91-1.06)	1.00	(0.93-1.08)
Normal HbA1c	1.00	ref	1.00	ref	1.00	ref	1.00	ref	1.00	ref
Pre-diabetes	1.65	(1.44-1.91)	1.43	(1.24-1.64)	1.39	(1.20-1.60)	1.22	(1.06-1.41)	1.14	(0.99-1.32)
Undiagnosed diabetes	1.33	(0.91-1.93)	1.26	(0.87-1.84)	1.22	(0.84-1.77)	1.01	(0.70-1.47)	0.95	(0.65-1.39)
Diagnosed diabetes	2.28	(2.01-2.59)	2.02	(1.78-2.30)	1.94	(1.71-2.20)	1.63	(1.43-1.86)	1.43	(1.25-1.64)
p-interaction	*0.0990*		*0.3739*		*0.4397*		*0.6282*		*0.6730*	
**Heart failure**										
Men										
Low-normal HbA1c	0.62	(0.59-0.66)	0.78	(0.74-0.83)	0.79	(0.75-0.84)	0.93	(0.88-0.98)	1.00	(0.94-1.05)
Normal HbA1c	1.00	ref	1.00	ref	1.00	ref	1.00	ref	1.00	ref
Pre-diabetes	1.91	(1.73-2.10)	1.65	(1.50-1.82)	1.60	(1.45-1.76)	1.20	(1.09-1.33)	1.08	(0.98-1.19)
Undiagnosed diabetes	1.99	(1.67-2.36)	1.96	(1.65-2.33)	1.86	(1.56-2.21)	1.21	(1.01-1.44)	1.15	(0.96-1.37)
Diagnosed diabetes	3.02	(2.83-3.22)	2.51	(2.35-2.68)	2.40	(2.24-2.56)	1.69	(1.58-1.81)	1.27	(1.18-1.36)
Women										
Low-normal HbA1c	0.60	(0.56-0.65)	0.88	(0.82-0.94)	0.89	(0.83-0.95)	1.04	(0.97-1.12)	1.09	(1.01-1.17)
Normal HbA1c	1.00	ref	1.00	ref	1.00	ref	1.00	ref	1.00	ref
Pre-diabetes	2.43	(2.16-2.73)	2.07	(1.84-2.32)	2.00	(1.78-2.25)	1.43	(1.27-1.61)	1.25	(1.11-1.41)
Undiagnosed diabetes	2.27	(1.71-3.01)	2.16	(1.62-2.86)	2.05	(1.55-2.73)	1.19	(0.89-1.58)	1.05	(0.79-1.40)
Diagnosed diabetes	4.19	(3.80-4.61)	3.69	(3.35-4.07)	3.46	(3.14-3.82)	1.99	(1.80-2.21)	1.44	(1.30-1.60)
p-interaction	*<0.0001*		*<0.0001*		*<0.0001*		*0.0114*		*0.0712*	

*A composite measure of all examined outcomes

Abbreviations: HbA1c, glycated haemoglobin; HR, hazard ratio; CI, confidence interval

Notes: Categories were defined by baseline HbA1c levels as follows: low-normal (<35 mmol/mol or <5.5%), normal (35-41 mmol/mol or 5.5-5.9%), pre-diabetes (42-47 mmol/mol or 6.0-6.4%), undiagnosed diabetes (≥48 mmol/mol or ≥6.5%), or diagnosed diabetes. Sex-specific hazard ratios from Cox proportional hazards models, adjusted for age at study entry, socio-demographics (i.e., ethnicity, deprivation), lifestyle characteristics (i.e., smoking status, alcohol consumption, physical activity, body mass index, waist-hip ratio, processed meat and fruit and vegetable intake), and clinical characteristics (i.e., total cholesterol, estimated glomerular filtration rate, C-reactive protein, diagnosed hypertension, use of antihypertensive medication or statins, and family history of cardiovascular disease).

eTable 5. Sensitivity analysis excluding events occurring within 180 days of follow-up

	Unadjusted		Age-adjusted		+ Socio-demographics		+ Lifestyle characteristics		+ Clinical characteristics	
	**HR**	**(95% CI)**	**HR**	**(95% CI)**	**HR**	**(95% CI)**	**HR**	**(95% CI)**	**HR**	**(95% CI)**
**Any cardiovascular disease***										
Men										
Low-normal HbA1c	0.72	(0.70-0.74)	0.86	(0.84-0.88)	0.87	(0.84-0.89)	0.95	(0.93-0.97)	0.98	(0.96-1.01)
Normal HbA1c	1.00	ref	1.00	ref	1.00	ref	1.00	ref	1.00	ref
Pre-diabetes	1.46	(1.38-1.54)	1.30	(1.23-1.37)	1.29	(1.22-1.36)	1.08	(1.02-1.14)	1.04	(0.98-1.09)
Undiagnosed diabetes	1.31	(1.18-1.46)	1.30	(1.17-1.44)	1.28	(1.15-1.42)	0.99	(0.89-1.10)	0.97	(0.87-1.07)
Diagnosed diabetes	1.81	(1.74-1.88)	1.54	(1.48-1.61)	1.52	(1.46-1.58)	1.26	(1.20-1.31)	1.06	(1.02-1.11)
Women										
Low-normal HbA1c	0.64	(0.62-0.65)	0.86	(0.84-0.89)	0.87	(0.84-0.89)	0.96	(0.93-0.99)	0.98	(0.95-1.01)
Normal HbA1c	1.00	ref	1.00	ref	1.00	ref	1.00	ref	1.00	ref
Pre-diabetes	1.63	(1.53-1.73)	1.43	(1.35-1.53)	1.43	(1.34-1.52)	1.15	(1.08-1.23)	1.08	(1.02-1.15)
Undiagnosed diabetes	1.33	(1.13-1.57)	1.28	(1.09-1.51)	1.27	(1.08-1.49)	0.90	(0.76-1.06)	0.85	(0.73-1.00)
Diagnosed diabetes	2.18	(2.06-2.31)	1.99	(1.88-2.11)	1.94	(1.83-2.06)	1.39	(1.31-1.47)	1.17	(1.10-1.24)
p-interaction	*<0.0001*		*<0.0001*		*<0.0001*		*0.0315*		*0.0464*	
**Coronary artery disease**										
Men										
Low-normal HbA1c	0.65	(0.61-0.68)	0.75	(0.71-0.79)	0.75	(0.71-0.79)	0.85	(0.80-0.89)	0.90	(0.86-0.95)
Normal HbA1c	1.00	ref	1.00	ref	1.00	ref	1.00	ref	1.00	ref
Pre-diabetes	1.58	(1.43-1.75)	1.44	(1.30-1.59)	1.41	(1.28-1.57)	1.20	(1.09-1.33)	1.15	(1.03-1.27)
Undiagnosed diabetes	1.76	(1.47-2.10)	1.73	(1.45-2.07)	1.69	(1.41-2.01)	1.37	(1.15-1.64)	1.32	(1.11-1.58)
Diagnosed diabetes	2.09	(1.94-2.24)	1.84	(1.71-1.98)	1.78	(1.66-1.91)	1.54	(1.43-1.66)	1.51	(1.40-1.64)
Women										
Low-normal HbA1c	0.56	(0.52-0.61)	0.71	(0.66-0.77)	0.72	(0.66-0.77)	0.83	(0.76-0.90)	0.89	(0.82-0.97)
Normal HbA1c	1.00	ref	1.00	ref	1.00	ref	1.00	ref	1.00	ref
Pre-diabetes	1.69	(1.45-1.96)	1.52	(1.31-1.77)	1.51	(1.30-1.75)	1.19	(1.02-1.38)	1.12	(0.96-1.30)
Undiagnosed diabetes	1.55	(1.07-2.24)	1.50	(1.04-2.16)	1.45	(1.01-2.10)	1.03	(0.71-1.49)	0.97	(0.67-1.41)
Diagnosed diabetes	2.76	(2.44-3.12)	2.55	(2.26-2.89)	2.46	(2.18-2.79)	1.78	(1.57-2.03)	1.79	(1.57-2.04)
p-interaction	*<0.0001*		*0.0001*		*0.0001*		*0.1394*		*0.0763*	
**Atrial fibrillation**										
Men										
Low-normal HbA1c	0.75	(0.72-0.77)	0.94	(0.91-0.98)	0.94	(0.91-0.98)	1.02	(0.98-1.06)	1.04	(1.01-1.08)
Normal HbA1c	1.00	ref	1.00	ref	1.00	ref	1.00	ref	1.00	ref
Pre-diabetes	1.50	(1.39-1.62)	1.29	(1.20-1.40)	1.30	(1.21-1.41)	1.09	(1.01-1.18)	1.03	(0.95-1.11)
Undiagnosed diabetes	1.36	(1.17-1.57)	1.34	(1.15-1.55)	1.35	(1.17-1.56)	1.01	(0.87-1.17)	1.00	(0.86-1.16)
Diagnosed diabetes	1.92	(1.82-2.03)	1.58	(1.50-1.67)	1.59	(1.50-1.68)	1.26	(1.19-1.33)	1.02	(0.96-1.08)
Women										
Low-normal HbA1c	0.66	(0.63-0.70)	0.98	(0.94-1.03)	0.98	(0.93-1.02)	1.06	(1.01-1.11)	1.06	(1.01-1.11)
Normal HbA1c	1.00	ref	1.00	ref	1.00	ref	1.00	ref	1.00	ref
Pre-diabetes	1.55	(1.41-1.71)	1.32	(1.20-1.45)	1.34	(1.21-1.47)	1.10	(0.99-1.21)	1.02	(0.92-1.12)
Undiagnosed diabetes	1.25	(0.97-1.61)	1.19	(0.92-1.53)	1.21	(0.94-1.56)	0.87	(0.67-1.12)	0.82	(0.64-1.06)
Diagnosed diabetes	2.14	(1.97-2.34)	1.89	(1.73-2.06)	1.89	(1.74-2.06)	1.34	(1.23-1.46)	1.06	(0.96-1.16)
p-interaction	*<0.0001*		*0.0131*		*0.0155*		*0.4301*		*0.6141*	
**Deep vein thrombosis**										
Men										
Low-normal HbA1c	0.84	(0.77-0.93)	0.97	(0.88-1.06)	0.97	(0.88-1.06)	1.07	(0.97-1.17)	1.06	(0.97-1.17)
Normal HbA1c	1.00	ref	1.00	ref	1.00	ref	1.00	ref	1.00	ref
Pre-diabetes	1.14	(0.91-1.43)	1.05	(0.84-1.31)	1.04	(0.83-1.30)	0.87	(0.69-1.09)	0.87	(0.69-1.09)
Undiagnosed diabetes	1.17	(0.77-1.77)	1.16	(0.76-1.75)	1.14	(0.75-1.72)	0.86	(0.57-1.31)	0.87	(0.57-1.31)
Diagnosed diabetes	1.44	(1.23-1.69)	1.28	(1.09-1.50)	1.27	(1.08-1.49)	1.02	(0.87-1.20)	1.06	(0.89-1.25)
Women										
Low-normal HbA1c	0.66	(0.59-0.73)	0.82	(0.74-0.91)	0.82	(0.74-0.92)	0.94	(0.84-1.04)	0.93	(0.84-1.04)
Normal HbA1c	1.00	ref	1.00	ref	1.00	ref	1.00	ref	1.00	ref
Pre-diabetes	1.38	(1.11-1.73)	1.26	(1.00-1.58)	1.25	(0.99-1.56)	0.97	(0.78-1.22)	0.97	(0.77-1.21)
Undiagnosed diabetes	1.34	(0.77-2.32)	1.30	(0.75-2.25)	1.29	(0.75-2.24)	0.87	(0.50-1.51)	0.87	(0.50-1.51)
Diagnosed diabetes	1.56	(1.25-1.94)	1.44	(1.16-1.80)	1.42	(1.14-1.77)	0.96	(0.77-1.21)	0.99	(0.78-1.24)
p-interaction	*0.0013*		*0.0461*		*0.0506*		*0.3370*		*0.3337*	
**Pulmonary embolism**										
Men										
Low-normal HbA1c	0.76	(0.70-0.82)	0.88	(0.81-0.95)	0.88	(0.81-0.95)	0.98	(0.91-1.06)	0.99	(0.92-1.07)
Normal HbA1c	1.00	ref	1.00	ref	1.00	ref	1.00	ref	1.00	ref
Pre-diabetes	1.48	(1.26-1.73)	1.34	(1.14-1.57)	1.33	(1.13-1.56)	1.08	(0.92-1.27)	1.07	(0.91-1.26)
Undiagnosed diabetes	1.32	(0.97-1.80)	1.31	(0.96-1.78)	1.29	(0.94-1.76)	0.93	(0.68-1.28)	0.92	(0.68-1.26)
Diagnosed diabetes	1.37	(1.20-1.56)	1.21	(1.06-1.37)	1.19	(1.05-1.36)	0.92	(0.81-1.05)	0.99	(0.86-1.14)
Women										
Low-normal HbA1c	0.60	(0.56-0.65)	0.77	(0.71-0.83)	0.77	(0.71-0.83)	0.88	(0.81-0.96)	0.89	(0.82-0.97)
Normal HbA1c	1.00	ref	1.00	ref	1.00	ref	1.00	ref	1.00	ref

Pre-diabetes	1.53	(1.30-1.80)	1.38	(1.17-1.63)	1.37	(1.16-1.61)	1.05	(0.89-1.24)	1.03	(0.87-1.22)
Undiagnosed diabetes	1.12	(0.71-1.76)	1.08	(0.69-1.70)	1.07	(0.68-1.69)	0.70	(0.44-1.10)	0.69	(0.44-1.08)
Diagnosed diabetes	1.63	(1.38-1.92)	1.51	(1.28-1.78)	1.48	(1.25-1.74)	0.97	(0.82-1.15)	1.02	(0.86-1.22)
p-interaction	*0.0001*		*0.0110*		*0.0133*		*0.2768*		*0.3009*	
**Stroke**										
Men										
Low-normal HbA1c	0.71	(0.67-0.76)	0.87	(0.82-0.93)	0.88	(0.83-0.93)	0.95	(0.89-1.01)	0.98	(0.92-1.04)
Normal HbA1c	1.00	ref	1.00	ref	1.00	ref	1.00	ref	1.00	ref
Pre-diabetes	1.45	(1.28-1.65)	1.27	(1.12-1.44)	1.24	(1.09-1.41)	1.10	(0.97-1.26)	1.05	(0.92-1.20)
Undiagnosed diabetes	1.49	(1.18-1.89)	1.47	(1.16-1.86)	1.42	(1.12-1.80)	1.21	(0.95-1.53)	1.17	(0.93-1.48)
Diagnosed diabetes	2.14	(1.96-2.33)	1.80	(1.65-1.96)	1.75	(1.60-1.91)	1.58	(1.44-1.73)	1.42	(1.29-1.56)
Women										
Low-normal HbA1c	0.66	(0.61-0.70)	0.91	(0.85-0.98)	0.92	(0.86-0.99)	0.99	(0.92-1.06)	1.01	(0.94-1.08)
Normal HbA1c	1.00	ref	1.00	ref	1.00	ref	1.00	ref	1.00	ref
Pre-diabetes	1.66	(1.43-1.91)	1.43	(1.24-1.64)	1.39	(1.20-1.60)	1.22	(1.06-1.41)	1.14	(0.99-1.32)
Undiagnosed diabetes	1.30	(0.89-1.91)	1.24	(0.85-1.82)	1.20	(0.82-1.75)	0.99	(0.68-1.45)	0.93	(0.64-1.37)
Diagnosed diabetes	2.28	(2.00-2.59)	2.02	(1.77-2.30)	1.93	(1.70-2.20)	1.62	(1.42-1.85)	1.43	(1.25-1.64)
p-interaction	*0.1244*		*0.3902*		*0.4571*		*0.6503*		*0.6971*	
**Heart failure**										
Men										
Low-normal HbA1c	0.62	(0.59-0.65)	0.78	(0.74-0.82)	0.79	(0.75-0.83)	0.92	(0.87-0.98)	0.99	(0.94-1.05)
Normal HbA1c	1.00	ref	1.00	ref	1.00	ref	1.00	ref	1.00	ref
Pre-diabetes	1.90	(1.72-2.10)	1.64	(1.49-1.81)	1.59	(1.44-1.75)	1.20	(1.09-1.32)	1.08	(0.98-1.19)
Undiagnosed diabetes	1.98	(1.66-2.36)	1.96	(1.64-2.33)	1.86	(1.56-2.21)	1.21	(1.01-1.44)	1.15	(0.96-1.37)
Diagnosed diabetes	3.01	(2.82-3.21)	2.50	(2.34-2.67)	2.39	(2.23-2.55)	1.69	(1.58-1.81)	1.27	(1.18-1.36)
Women										
Low-normal HbA1c	0.60	(0.56-0.65)	0.88	(0.82-0.94)	0.89	(0.82-0.95)	1.04	(0.97-1.12)	1.08	(1.01-1.17)
Normal HbA1c	1.00	ref	1.00	ref	1.00	ref	1.00	ref	1.00	ref
Pre-diabetes	2.43	(2.16-2.73)	2.06	(1.83-2.32)	2.00	(1.78-2.25)	1.43	(1.27-1.61)	1.25	(1.11-1.41)
Undiagnosed diabetes	2.29	(1.72-3.04)	2.17	(1.64-2.89)	2.07	(1.56-2.75)	1.20	(0.90-1.60)	1.07	(0.80-1.42)
Diagnosed diabetes	4.15	(3.76-4.58)	3.66	(3.31-4.03)	3.43	(3.11-3.79)	1.98	(1.79-2.19)	1.44	(1.29-1.60)
p-interaction	*<0.0001*		*<0.0001*		*<0.0001*		*0.0124*		*0.0740*	

*A composite measure of all examined outcomes

Abbreviations: HbA1c, glycated haemoglobin; HR, hazard ratio; CI, confidence interval

Notes: Categories were defined by baseline HbA1c levels as follows: low-normal (<35 mmol/mol or <5.5%), normal (35-41 mmol/mol or 5.5-5.9%), pre-diabetes (42-47 mmol/mol or 6.0-6.4%), undiagnosed diabetes (≥48 mmol/mol or ≥6.5%), or diagnosed diabetes. Sex-specific hazard ratios from Cox proportional hazards models, adjusted for age at study entry, socio-demographics (i.e., ethnicity, deprivation), lifestyle characteristics (i.e., smoking status, alcohol consumption, physical activity, body mass index, waist-hip ratio, processed meat and fruit and vegetable intake), and clinical characteristics (i.e., total cholesterol, estimated glomerular filtration rate, C-reactive protein, diagnosed hypertension, use of antihypertensive medication or statins, and family history of cardiovascular disease).

eTable 6. Sensitivity analysis excluding individuals with diagnosed diabetes

	Unadjusted		Age-adjusted		+ Socio-demographics		+ Lifestyle characteristics		+ Clinical characteristics	
	**HR**	**(95% CI)**	**HR**	**(95% CI)**	**HR**	**(95% CI)**	**HR**	**(95% CI)**	**HR**	**(95% CI)**
**Any cardiovascular disease***										
Men										
Low-normal HbA1c	0.72	(0.70-0.74)	0.86	(0.84-0.89)	0.87	(0.85-0.89)	0.95	(0.93-0.97)	0.98	(0.96-1.01)
Normal HbA1c	1.00	ref	1.00	ref	1.00	ref	1.00	ref	1.00	ref
Pre-diabetes	1.46	(1.38-1.54)	1.3	(1.23-1.37)	1.29	(1.22-1.36)	1.09	(1.03-1.15)	1.04	(0.98-1.10)
Undiagnosed diabetes	1.32	(1.19-1.46)	1.31	(1.18-1.45)	1.29	(1.16-1.43)	1	(0.90-1.11)	0.98	(0.88-1.09)
Women										
Low-normal HbA1c	0.64	(0.62-0.65)	0.87	(0.84-0.89)	0.87	(0.84-0.90)	0.96	(0.93-0.99)	0.99	(0.96-1.02)
Normal HbA1c	1.00	ref	1.00	ref	1.00	ref	1.00	ref	1.00	ref
Pre-diabetes	1.67	(1.57-1.77)	1.47	(1.38-1.56)	1.46	(1.37-1.55)	1.18	(1.11-1.26)	1.11	(1.04-1.18)
Undiagnosed diabetes	1.38	(1.18-1.62)	1.33	(1.14-1.56)	1.32	(1.13-1.54)	0.93	(0.80-1.09)	0.89	(0.76-1.04)
p-interaction	*<0.0001*		*0.0380*		*0.0284*		*0.1844*		*0.2966*	
**Coronary artery disease**										
Men										
Low-normal HbA1c	0.64	(0.61-0.68)	0.75	(0.71-0.78)	0.75	(0.71-0.79)	0.84	(0.80-0.89)	0.90	(0.85-0.95)
Normal HbA1c	1.00	ref	1.00	ref	1.00	ref	1.00	ref	1.00	ref
Pre-diabetes	1.58	(1.43-1.75)	1.44	(1.30-1.59)	1.42	(1.28-1.57)	1.22	(1.10-1.35)	1.16	(1.05-1.29)
Undiagnosed diabetes	1.76	(1.48-2.10)	1.74	(1.46-2.07)	1.70	(1.43-2.03)	1.41	(1.18-1.68)	1.35	(1.13-1.61)
Women										
Low-normal HbA1c	0.56	(0.52-0.60)	0.71	(0.65-0.77)	0.71	(0.66-0.77)	0.82	(0.76-0.89)	0.89	(0.82-0.96)
Normal HbA1c	1.00	ref	1.00	ref	1.00	ref	1.00	ref	1.00	ref
Pre-diabetes	1.70	(1.46-1.96)	1.53	(1.32-1.77)	1.52	(1.32-1.77)	1.20	(1.04-1.39)	1.13	(0.98-1.31)
Undiagnosed diabetes	1.57	(1.09-2.25)	1.52	(1.06-2.18)	1.49	(1.04-2.14)	1.07	(0.74-1.53)	1.00	(0.70-1.44)
p-interaction	*0.0092*		*0.4835*		*0.4419*		*0.5523*		*0.5083*	
**Atrial fibrillation**										
Men										
Low-normal HbA1c	0.75	(0.72-0.77)	0.95	(0.91-0.98)	0.94	(0.91-0.98)	1.02	(0.98-1.06)	1.04	(1.00-1.08)
Normal HbA1c	1.00	ref	1.00	ref	1.00	ref	1.00	ref	1.00	ref
Pre-diabetes	1.50	(1.40-1.62)	1.29	(1.20-1.40)	1.3	(1.21-1.41)	1.09	(1.01-1.18)	1.03	(0.96-1.12)
Undiagnosed diabetes	1.37	(1.18-1.58)	1.35	(1.17-1.56)	1.36	(1.18-1.57)	1.03	(0.89-1.19)	1.02	(0.88-1.18)
Women										
Low-normal HbA1c	0.67	(0.64-0.70)	0.99	(0.94-1.04)	0.99	(0.94-1.03)	1.06	(1.02-1.12)	1.06	(1.01-1.11)
Normal HbA1c	1.00	ref	1.00	ref	1.00	ref	1.00	ref	1.00	ref
Pre-diabetes	1.59	(1.45-1.74)	1.35	(1.22-1.48)	1.36	(1.24-1.50)	1.12	(1.02-1.23)	1.04	(0.95-1.15)
Undiagnosed diabetes	1.29	(1.01-1.65)	1.22	(0.95-1.56)	1.25	(0.98-1.60)	0.9	(0.70-1.15)	0.85	(0.66-1.09)
p-interaction	*0.0006*		*0.3974*		*0.4168*		*0.3750*		*0.5336*	
**Deep vein thrombosis**										
Men										
Low-normal HbA1c	0.85	(0.77-0.93)	0.97	(0.88-1.06)	0.97	(0.89-1.07)	1.07	(0.98-1.18)	1.07	(0.97-1.18)
Normal HbA1c	1.00	ref	1.00	ref	1.00	ref	1.00	ref	1.00	ref
Pre-diabetes	1.22	(0.98-1.51)	1.11	(0.89-1.38)	1.1	(0.89-1.37)	0.91	(0.73-1.14)	0.91	(0.73-1.14)
Undiagnosed diabetes	1.14	(0.76-1.73)	1.13	(0.75-1.71)	1.12	(0.74-1.69)	0.84	(0.55-1.27)	0.84	(0.55-1.27)
Women										
Low-normal HbA1c	0.67	(0.60-0.74)	0.84	(0.75-0.93)	0.84	(0.76-0.93)	0.95	(0.86-1.06)	0.95	(0.85-1.05)
Normal HbA1c	1.00	ref	1.00	ref	1.00	ref	1.00	ref	1.00	ref
Pre-diabetes	1.47	(1.18-1.83)	1.34	(1.08-1.66)	1.32	(1.06-1.64)	1.03	(0.83-1.28)	1.02	(0.82-1.27)
Undiagnosed diabetes	1.52	(0.91-2.53)	1.47	(0.88-2.45)	1.46	(0.88-2.43)	0.98	(0.59-1.64)	0.98	(0.58-1.63)
p-interaction	*0.0011*		*0.0473*		*0.0491*		*0.2250*		*0.2303*	
**Pulmonary embolism**										
Men										
Low-normal HbA1c	0.76	(0.70-0.82)	0.88	(0.82-0.95)	0.88	(0.82-0.95)	0.99	(0.91-1.06)	1	(0.92-1.08)
Normal HbA1c	1.00	ref	1.00	ref	1.00	ref	1.00	ref	1.00	ref
Pre-diabetes	1.47	(1.25-1.72)	1.33	(1.14-1.56)	1.32	(1.13-1.55)	1.07	(0.91-1.26)	1.07	(0.91-1.25)
Undiagnosed diabetes	1.33	(0.98-1.81)	1.32	(0.97-1.79)	1.30	(0.96-1.77)	0.94	(0.69-1.28)	0.93	(0.68-1.26)
Women										
Low-normal HbA1c	0.61	(0.56-0.66)	0.78	(0.72-0.85)	0.78	(0.72-0.85)	0.9	(0.83-0.98)	0.91	(0.84-0.99)
Normal HbA1c	1.00	ref	1.00	ref	1.00	ref	1.00	ref	1.00	ref
Pre-diabetes	1.59	(1.36-1.87)	1.44	(1.23-1.69)	1.42	(1.21-1.67)	1.09	(0.92-1.28)	1.07	(0.91-1.26)
Undiagnosed diabetes	1.17	(0.75-1.81)	1.13	(0.72-1.75)	1.12	(0.72-1.74)	0.73	(0.47-1.13)	0.71	(0.46-1.11)
p-interaction	*0.0007*		*0.1135*		*0.1139*		*0.3127*		*0.3135*	
**Stroke**										
Men										

Low-normal HbA1c	0.71	(0.67-0.75)	0.87	(0.82-0.93)	0.88	(0.83-0.93)	0.95	(0.89-1.01)	0.98	(0.92-1.04)
Normal HbA1c	1.00	ref	1.00	ref	1.00	ref	1.00	ref	1.00	ref
Pre-diabetes	1.44	(1.27-1.64)	1.26	(1.10-1.43)	1.23	(1.08-1.40)	1.1	(0.96-1.25)	1.04	(0.92-1.19)
Undiagnosed diabetes	1.52	(1.21-1.91)	1.50	(1.19-1.88)	1.45	(1.15-1.83)	1.24	(0.98-1.56)	1.20	(0.95-1.52)
Women										
Low-normal HbA1c	0.65	(0.61-0.70)	0.92	(0.85-0.98)	0.92	(0.86-0.99)	0.99	(0.92-1.06)	1.01	(0.94-1.08)
Normal HbA1c	1.00	ref	1.00	ref	1.00	ref	1.00	ref	1.00	ref
Pre-diabetes	1.65	(1.44-1.91)	1.42	(1.23-1.63)	1.39	(1.20-1.60)	1.23	(1.06-1.41)	1.15	(0.99-1.33)
Undiagnosed diabetes	1.33	(0.91-1.93)	1.26	(0.87-1.83)	1.22	(0.84-1.78)	1.02	(0.70-1.49)	0.96	(0.66-1.40)
p-interaction		*0.1015*		*0.3903*		*0.4020*		*0.4386*		*0.4923*
**Heart failure**										
Men										
Low-normal HbA1c	0.62	(0.59-0.66)	0.79	(0.74-0.83)	0.8	(0.75-0.84)	0.93	(0.88-0.98)	1	(0.94-1.06)
Normal HbA1c	1.00	ref	1.00	ref	1.00	ref	1.00	ref	1.00	ref
Pre-diabetes	1.91	(1.73-2.11)	1.65	(1.49-1.81)	1.59	(1.44-1.76)	1.2	(1.09-1.32)	1.08	(0.97-1.19)
Undiagnosed diabetes	1.99	(1.67-2.36)	1.96	(1.65-2.33)	1.86	(1.56-2.22)	1.2	(1.00-1.43)	1.15	(0.96-1.37)
Women										
Low-normal HbA1c	0.60	(0.56-0.65)	0.89	(0.83-0.95)	0.9	(0.83-0.96)	1.05	(0.98-1.13)	1.09	(1.02-1.18)
Normal HbA1c	1.00	ref	1.00	ref	1.00	ref	1.00	ref	1.00	ref
Pre-diabetes	2.43	(2.16-2.73)	2.05	(1.83-2.31)	1.99	(1.77-2.24)	1.41	(1.25-1.59)	1.24	(1.10-1.40)
Undiagnosed diabetes	2.27	(1.71-3.01)	2.15	(1.62-2.86)	2.05	(1.55-2.73)	1.17	(0.88-1.56)	1.04	(0.78-1.38)
p-interaction		*0.0080*		*0.0064*		*0.0064*		*0.0211*		*0.0840*

*A composite measure of all examined outcomes

Abbreviations: HbA1c, glycated haemoglobin; HR, hazard ratio; CI, confidence interval

Notes: Categories were defined by baseline HbA1c levels as follows: low-normal (<35 mmol/mol or <5.5%), normal (35-41 mmol/mol or 5.5-5.9%), pre-diabetes (42-47 mmol/mol or 6.0-6.4%), or undiagnosed diabetes (≥48 mmol/mol or ≥6.5%). Sex-specific hazard ratios from Cox proportional hazards models, adjusted for age at study entry, socio-demographics (i.e., ethnicity, deprivation), lifestyle characteristics (i.e., smoking status, alcohol consumption, physical activity, body mass index, waist-hip ratio, processed meat and fruit and vegetable intake), and clinical characteristics (i.e., total cholesterol, estimated glomerular filtration rate, C-reactive protein, diagnosed hypertension, use of antihypertensive medication or statins, and family history of cardiovascular disease).

eTable 7. Sensitivity analysis excluding individuals with any cardiovascular disease at baseline instead of outcome-specific exclusion

	**Unadjusted**		**Age-adjusted**		**+ Socio-demographics**		**+ Lifestyle characteristics**		**+ Clinical characteristics**	
	**HR**	**(95% CI)**	**HR**	**(95% CI)**	**HR**	**(95% CI)**	**HR**	**(95% CI)**	**HR**	**(95% CI)**
**Any cardiovascular disease***										
Men										
Low-normal HbA1c	0.72	(0.70-0.74)	0.86	(0.84-0.88)	0.87	(0.84-0.89)	0.95	(0.92-0.97)	0.98	(0.96-1.01)
Normal HbA1c	1.00	ref	1.00	ref	1.00	ref	1.00	ref	1.00	ref
Pre-diabetes	1.46	(1.38-1.54)	1.30	(1.24-1.38)	1.29	(1.22-1.36)	1.09	(1.03-1.15)	1.04	(0.98-1.10)
Undiagnosed diabetes	1.32	(1.19-1.46)	1.31	(1.18-1.45)	1.28	(1.16-1.42)	1.00	(0.90-1.11)	0.97	(0.88-1.08)
Diagnosed diabetes	1.82	(1.75-1.89)	1.55	(1.49-1.61)	1.53	(1.47-1.59)	1.26	(1.21-1.32)	1.06	(1.02-1.11)
Women										
Low-normal HbA1c	0.64	(0.62-0.65)	0.86	(0.84-0.89)	0.87	(0.84-0.89)	0.96	(0.93-0.99)	0.99	(0.96-1.02)
Normal HbA1c	1.00	ref	1.00	ref	1.00	ref	1.00	ref	1.00	ref
Pre-diabetes	1.67	(1.57-1.77)	1.47	(1.38-1.56)	1.46	(1.38-1.55)	1.18	(1.11-1.26)	1.11	(1.04-1.18)
Undiagnosed diabetes	1.38	(1.18-1.62)	1.33	(1.14-1.56)	1.32	(1.12-1.54)	0.93	(0.79-1.09)	0.88	(0.75-1.03)
Diagnosed diabetes	2.19	(2.07-2.32)	2.00	(1.89-2.12)	1.95	(1.84-2.07)	1.40	(1.32-1.48)	1.17	(1.10-1.24)
p-interaction		*<0.0001*		*<0.0001*		*<0.0001*		*0.0197*		*0.0387*
**Coronary artery disease**										
Men										
Low-normal HbA1c	0.66	(0.63-0.70)	0.76	(0.72-0.80)	0.76	(0.72-0.80)	0.86	(0.81-0.90)	0.91	(0.86-0.96)
Normal HbA1c	1.00	ref	1.00	ref	1.00	ref	1.00	ref	1.00	ref
Pre-diabetes	1.52	(1.36-1.70)	1.39	(1.25-1.56)	1.38	(1.23-1.54)	1.18	(1.05-1.32)	1.15	(1.02-1.28)
Undiagnosed diabetes	1.70	(1.40-2.07)	1.69	(1.39-2.05)	1.66	(1.36-2.01)	1.37	(1.13-1.66)	1.32	(1.08-1.60)
Diagnosed diabetes	1.83	(1.68-1.98)	1.62	(1.49-1.76)	1.58	(1.46-1.72)	1.40	(1.28-1.52)	1.55	(1.41-1.70)
Women										
Low-normal HbA1c	0.56	(0.52-0.61)	0.70	(0.65-0.76)	0.71	(0.65-0.77)	0.82	(0.75-0.89)	0.88	(0.81-0.96)
Normal HbA1c	1.00	ref	1.00	ref	1.00	ref	1.00	ref	1.00	ref
Pre-diabetes	1.55	(1.32-1.83)	1.41	(1.20-1.66)	1.40	(1.19-1.65)	1.11	(0.94-1.30)	1.06	(0.90-1.25)
Undiagnosed diabetes	1.44	(0.96-2.15)	1.39	(0.93-2.08)	1.36	(0.91-2.04)	0.97	(0.65-1.45)	0.92	(0.61-1.37)
Diagnosed diabetes	2.46	(2.14-2.82)	2.29	(1.99-2.63)	2.23	(1.94-2.56)	1.63	(1.42-1.88)	1.84	(1.59-2.12)
p-interaction		*<0.0001*		*0.0001*		*0.0001*		*0.0888*		*0.0698*
**Atrial fibrillation**										
Men										
Low-normal HbA1c	0.76	(0.73-0.79)	0.96	(0.92-1.00)	0.96	(0.92-0.99)	1.03	(0.99-1.07)	1.04	(1.00-1.08)
Normal HbA1c	1.00	ref	1.00	ref	1.00	ref	1.00	ref	1.00	ref
Pre-diabetes	1.46	(1.34-1.59)	1.27	(1.17-1.38)	1.28	(1.18-1.39)	1.07	(0.99-1.17)	1.03	(0.95-1.12)
Undiagnosed diabetes	1.27	(1.08-1.49)	1.26	(1.07-1.48)	1.28	(1.09-1.51)	0.96	(0.82-1.13)	0.95	(0.81-1.12)
Diagnosed diabetes	1.82	(1.71-1.93)	1.49	(1.41-1.58)	1.51	(1.42-1.60)	1.21	(1.13-1.28)	0.99	(0.93-1.06)
Women										
Low-normal HbA1c	0.68	(0.65-0.72)	1.01	(0.96-1.06)	1.00	(0.96-1.05)	1.08	(1.03-1.13)	1.08	(1.03-1.13)
Normal HbA1c	1.00	ref	1.00	ref	1.00	ref	1.00	ref	1.00	ref
Pre-diabetes	1.56	(1.41-1.73)	1.33	(1.20-1.47)	1.35	(1.22-1.50)	1.12	(1.01-1.24)	1.05	(0.95-1.16)
Undiagnosed diabetes	1.23	(0.94-1.60)	1.16	(0.89-1.52)	1.20	(0.92-1.57)	0.86	(0.66-1.12)	0.82	(0.63-1.07)
Diagnosed diabetes	1.99	(1.81-2.18)	1.76	(1.60-1.93)	1.77	(1.61-1.95)	1.26	(1.14-1.39)	1.01	(0.92-1.12)
p-interaction		*0.0002*		*0.0500*		*0.0531*		*0.4964*		*0.6867*
**Deep vein thrombosis**										
Men										
Low-normal HbA1c	0.87	(0.79-0.95)	0.99	(0.89-1.09)	0.99	(0.90-1.09)	1.09	(0.99-1.20)	1.08	(0.98-1.20)
Normal HbA1c	1.00	ref	1.00	ref	1.00	ref	1.00	ref	1.00	ref
Pre-diabetes	1.17	(0.92-1.49)	1.07	(0.84-1.37)	1.07	(0.84-1.36)	0.88	(0.69-1.13)	0.89	(0.70-1.13)
Undiagnosed diabetes	1.12	(0.71-1.76)	1.11	(0.71-1.75)	1.10	(0.70-1.74)	0.83	(0.52-1.30)	0.83	(0.52-1.31)
Diagnosed diabetes	1.53	(1.29-1.81)	1.36	(1.15-1.62)	1.36	(1.14-1.61)	1.09	(0.91-1.29)	1.17	(0.98-1.41)
Women										
Low-normal HbA1c	0.68	(0.61-0.75)	0.84	(0.76-0.94)	0.84	(0.76-0.94)	0.95	(0.85-1.06)	0.95	(0.85-1.06)
Normal HbA1c	1.00	ref	1.00	ref	1.00	ref	1.00	ref	1.00	ref
Pre-diabetes	1.44	(1.14-1.81)	1.31	(1.04-1.65)	1.30	(1.03-1.64)	1.02	(0.81-1.29)	1.01	(0.80-1.28)
Undiagnosed diabetes	1.13	(0.60-2.10)	1.10	(0.59-2.04)	1.09	(0.59-2.04)	0.74	(0.39-1.38)	0.74	(0.39-1.38)
Diagnosed diabetes	1.57	(1.25-1.99)	1.47	(1.17-1.86)	1.45	(1.15-1.83)	0.99	(0.78-1.25)	1.04	(0.81-1.33)
p-interaction		*0.0029*		*0.0836*		*0.0890*		*0.2978*		*0.2920*
**Pulmonary embolism**										
Men										
Low-normal HbA1c	0.77	(0.71-0.83)	0.89	(0.82-0.96)	0.89	(0.82-0.96)	0.99	(0.91-1.07)	1.00	(0.92-1.08)
Normal HbA1c	1.00	ref	1.00	ref	1.00	ref	1.00	ref	1.00	ref
Pre-diabetes	1.44	(1.21-1.72)	1.32	(1.11-1.57)	1.31	(1.10-1.56)	1.05	(0.88-1.26)	1.05	(0.88-1.26)
Undiagnosed diabetes	1.42	(1.03-1.96)	1.41	(1.02-1.95)	1.40	(1.02-1.94)	1.01	(0.73-1.39)	0.99	(0.72-1.37)
Diagnosed diabetes	1.33	(1.15-1.53)	1.17	(1.01-1.35)	1.16	(1.01-1.34)	0.90	(0.78-1.04)	1.00	(0.86-1.17)
Women										
Low-normal HbA1c	0.64	(0.59-0.69)	0.81	(0.75-0.88)	0.81	(0.75-0.89)	0.93	(0.86-1.02)	0.94	(0.87-1.03)
Normal HbA1c	1.00	ref	1.00	ref	1.00	ref	1.00	ref	1.00	ref

Pre-diabetes	1.58	(1.33-1.87)	1.43	(1.20-1.69)	1.42	(1.20-1.68)	1.08	(0.91-1.29)	1.07	(0.90-1.27)
Undiagnosed diabetes	1.26	(0.80-1.98)	1.22	(0.78-1.92)	1.22	(0.78-1.92)	0.79	(0.50-1.25)	0.78	(0.50-1.23)
Diagnosed diabetes	1.64	(1.38-1.96)	1.53	(1.28-1.82)	1.51	(1.26-1.79)	0.99	(0.83-1.19)	1.08	(0.89-1.30)
p-interaction	*0.0010*		*0.0354*		*0.0418*		*0.5669*		*0.6431*	
**Stroke**										
Men										
Low-normal HbA1c	0.73	(0.69-0.78)	0.89	(0.83-0.95)	0.90	(0.84-0.96)	0.97	(0.91-1.03)	0.99	(0.93-1.06)
Normal HbA1c	1.00	ref	1.00	ref	1.00	ref	1.00	ref	1.00	ref
Pre-diabetes	1.42	(1.22-1.64)	1.25	(1.08-1.45)	1.22	(1.05-1.41)	1.09	(0.94-1.26)	1.05	(0.91-1.22)
Undiagnosed diabetes	1.43	(1.10-1.86)	1.42	(1.09-1.85)	1.37	(1.06-1.79)	1.18	(0.90-1.53)	1.14	(0.87-1.49)
Diagnosed diabetes	2.01	(1.82-2.22)	1.69	(1.53-1.86)	1.64	(1.49-1.81)	1.51	(1.36-1.67)	1.40	(1.26-1.56)
Women										
Low-normal HbA1c	0.67	(0.62-0.72)	0.92	(0.86-0.99)	0.93	(0.86-1.00)	1.00	(0.93-1.08)	1.02	(0.94-1.10)
Normal HbA1c	1.00	ref	1.00	ref	1.00	ref	1.00	ref	1.00	ref
Pre-diabetes	1.63	(1.40-1.90)	1.41	(1.21-1.65)	1.38	(1.18-1.60)	1.22	(1.05-1.42)	1.16	(0.99-1.35)
Undiagnosed diabetes	1.15	(0.75-1.77)	1.09	(0.71-1.68)	1.06	(0.69-1.62)	0.88	(0.57-1.36)	0.84	(0.54-1.29)
Diagnosed diabetes	2.14	(1.86-2.46)	1.91	(1.66-2.20)	1.83	(1.59-2.11)	1.56	(1.34-1.80)	1.43	(1.23-1.66)
p-interaction	*0.0843*		*0.3685*		*0.4204*		*0.5943*		*0.6339*	
**Heart failure**										
Men										
Low-normal HbA1c	0.66	(0.62-0.70)	0.83	(0.78-0.88)	0.84	(0.79-0.89)	0.98	(0.92-1.04)	1.03	(0.96-1.09)
Normal HbA1c	1.00	ref	1.00	ref	1.00	ref	1.00	ref	1.00	ref
Pre-diabetes	1.76	(1.57-1.99)	1.54	(1.37-1.73)	1.49	(1.32-1.68)	1.11	(0.99-1.25)	1.04	(0.92-1.17)
Undiagnosed diabetes	1.96	(1.60-2.41)	1.95	(1.59-2.39)	1.86	(1.51-2.28)	1.20	(0.98-1.48)	1.15	(0.93-1.41)
Diagnosed diabetes	2.79	(2.58-3.02)	2.31	(2.13-2.50)	2.22	(2.05-2.40)	1.60	(1.47-1.73)	1.27	(1.17-1.39)
Women										
Low-normal HbA1c	0.63	(0.59-0.68)	0.91	(0.85-0.99)	0.92	(0.85-1.00)	1.09	(1.01-1.18)	1.12	(1.04-1.21)
Normal HbA1c	1.00	ref	1.00	ref	1.00	ref	1.00	ref	1.00	ref
Pre-diabetes	2.26	(1.98-2.59)	1.93	(1.69-2.21)	1.87	(1.64-2.14)	1.33	(1.16-1.53)	1.21	(1.05-1.38)
Undiagnosed diabetes	2.19	(1.59-3.02)	2.08	(1.51-2.87)	1.99	(1.44-2.75)	1.14	(0.83-1.58)	1.03	(0.74-1.42)
Diagnosed diabetes	3.83	(3.42-4.29)	3.39	(3.03-3.80)	3.19	(2.85-3.58)	1.84	(1.63-2.07)	1.43	(1.27-1.62)
p-interaction	*<0.0001*		*<0.0001*		*<0.0001*		*0.0667*		*0.1510*	

*A composite measure of all examined outcomes

Abbreviations: HbA1c, glycated haemoglobin; HR, hazard ratio; CI, confidence interval

Notes: Categories were defined by baseline HbA1c levels as follows: low-normal (<35 mmol/mol or <5.5%), normal (35-41 mmol/mol or 5.5-5.9%), pre-diabetes (42-47 mmol/mol or 6.0-6.4%), undiagnosed diabetes (≥48 mmol/mol or ≥6.5%), or diagnosed diabetes. Sex-specific hazard ratios from Cox proportional hazards models, adjusted for age at study entry, socio-demographics (i.e., ethnicity, deprivation), lifestyle characteristics (i.e., smoking status, alcohol consumption, physical activity, body mass index, waist-hip ratio, processed meat and fruit and vegetable intake), and clinical characteristics (i.e., total cholesterol, estimated glomerular filtration rate, C-reactive protein, diagnosed hypertension, use of antihypertensive medication or statins, and family history of cardiovascular disease).

eTable 8. Sensitivity analysis using age as time scale in the Cox regression models

	Age-adjusted		+ Socio-demographics		+ Lifestyle characteristics		+ Clinical characteristics	
	**HR**	**(95% CI)**	**HR**	**(95% CI)**	**HR**	**(95% CI)**	**HR**	**(95% CI)**
**Any cardiovascular disease***								
Men								
Low-normal HbA1c	0.86	(0.84-0.88)	0.86	(0.84-0.89)	0.95	(0.92-0.97)	0.98	(0.96-1.01)
Normal HbA1c	1.00	ref	1.00	ref	1.00	ref	1.00	ref
Pre-diabetes	1.31	(1.24-1.38)	1.29	(1.22-1.36)	1.09	(1.03-1.15)	1.04	(0.98-1.09)
Undiagnosed diabetes	1.31	(1.18-1.45)	1.29	(1.16-1.43)	1.00	(0.90-1.11)	0.97	(0.88-1.08)
Diagnosed diabetes	1.56	(1.50-1.62)	1.53	(1.47-1.59)	1.27	(1.22-1.32)	1.06	(1.02-1.11)
Women								
Low-normal HbA1c	0.86	(0.84-0.89)	0.86	(0.84-0.89)	0.96	(0.93-0.99)	0.99	(0.96-1.02)
Normal HbA1c	1.00	ref	1.00	ref	1.00	ref	1.00	ref
Pre-diabetes	1.47	(1.39-1.57)	1.47	(1.38-1.56)	1.19	(1.12-1.26)	1.11	(1.04-1.18)
Undiagnosed diabetes	1.33	(1.14-1.56)	1.32	(1.12-1.54)	0.93	(0.79-1.09)	0.88	(0.75-1.03)
Diagnosed diabetes	2.01	(1.89-2.12)	1.96	(1.85-2.07)	1.40	(1.32-1.49)	1.17	(1.10-1.24)
p-interaction		*<0.0001*		*<0.0001*		*0.0186*		*0.0366*

*A composite measure of all examined outcomes.

Abbreviations: HbA1c, glycated haemoglobin; HR, hazard ratio; CI, confidence interval

Notes: Categories were defined by baseline HbA1c levels as follows: low-normal (<35 mmol/mol or <5.5%), normal (35-41 mmol/mol or 5.5-5.9%), pre-diabetes (42-47 mmol/mol or 6.0-6.4%), undiagnosed diabetes (≥48 mmol/mol or ≥6.5%), or diagnosed diabetes. Sex-specific hazard ratios from Cox proportional hazards models, adjusted for age at study entry, socio-demographics (i.e., ethnicity, deprivation), lifestyle characteristics (i.e., smoking status, alcohol consumption, physical activity, body mass index, waist-hip ratio, processed meat and fruit and vegetable intake), and clinical characteristics (i.e., total cholesterol, estimated glomerular filtration rate, C-reactive protein, diagnosed hypertension, use of antihypertensive medication or statins, and family history of cardiovascular disease).

eTable 9. Sensitivity analysis including sex-confounder interactions in the Cox regression model

	**Unadjusted**		**Age-adjusted**		**+ Socio-demographics**		**+ Lifestyle characteristics**		**+ Clinical characteristics**	
	**HR**	**(95% CI)**	**HR**	**(95% CI)**	**HR**	**(95% CI)**	**HR**	**(95% CI)**	**HR**	**(95% CI)**
**Any cardiovascular disease***										
Men										
Low-normal HbA1c	0.72	(0.70-0.74)	0.85	(0.83-0.87)	0.86	(0.83-0.88)	0.93	(0.91-0.95)	0.96	(0.94-0.99)
Normal HbA1c	1.00	ref	1.00	ref	1.00	ref	1.00	ref	1.00	ref
Pre-diabetes	1.46	(1.38-1.54)	1.31	(1.24-1.39)	1.31	(1.24-1.38)	1.11	(1.05-1.17)	1.06	(1.00-1.12)
Undiagnosed diabetes	1.32	(1.19-1.46)	1.31	(1.18-1.45)	1.30	(1.17-1.44)	1.02	(0.92-1.13)	0.99	(0.89-1.10)
Diagnosed diabetes	1.82	(1.75-1.89)	1.57	(1.50-1.63)	1.55	(1.49-1.61)	1.29	(1.23-1.34)	1.10	(1.06-1.15)
Women										
Low-normal HbA1c	0.64	(0.62-0.65)	0.88	(0.86-0.91)	0.89	(0.86-0.92)	0.99	(0.96-1.02)	1.02	(0.99-1.05)
Normal HbA1c	1.00	ref	1.00	ref	1.00	ref	1.00	ref	1.00	ref
Pre-diabetes	1.67	(1.57-1.77)	1.45	(1.37-1.54)	1.43	(1.34-1.52)	1.15	(1.08-1.22)	1.06	(1.00-1.13)
Undiagnosed diabetes	1.38	(1.18-1.62)	1.33	(1.13-1.55)	1.29	(1.10-1.51)	0.90	(0.77-1.06)	0.85	(0.73-1.00)
Diagnosed diabetes	2.19	(2.07-2.32)	1.98	(1.87-2.09)	1.91	(1.80-2.02)	1.35	(1.27-1.44)	1.05	(0.99-1.12)
p-interaction	*<0.0001*		*<0.0001*		*<0.0001*		*0.0110*		*0.0115*	

*A composite measure of all examined outcomes

Abbreviations: HbA1c, glycated haemoglobin; HR, hazard ratio; CI, confidence interval

Notes: Categories were defined by baseline HbA1c levels as follows: low-normal (<35 mmol/mol or <5.5%), normal (35-41 mmol/mol or 5.5-5.9%), pre-diabetes (42-47 mmol/mol or 6.0-6.4%), undiagnosed diabetes (≥48 mmol/mol or ≥6.5%), or diagnosed diabetes. Sex-specific hazard ratios from Cox proportional hazards models, adjusted for age at study entry, socio-demographics (i.e., ethnicity, deprivation), lifestyle characteristics (i.e., smoking status, alcohol consumption, physical activity, body mass index, waist-hip ratio, processed meat and fruit and vegetable intake), and clinical characteristics (i.e., total cholesterol, estimated glomerular filtration rate, C-reactive protein, diagnosed hypertension, use of antihypertensive medication or statins, and family history of cardiovascular disease).

eFigure 1. Schoenfeld residuals testing the proportional hazards assumption between HbA1c category and any cardiovascular disease, by sex

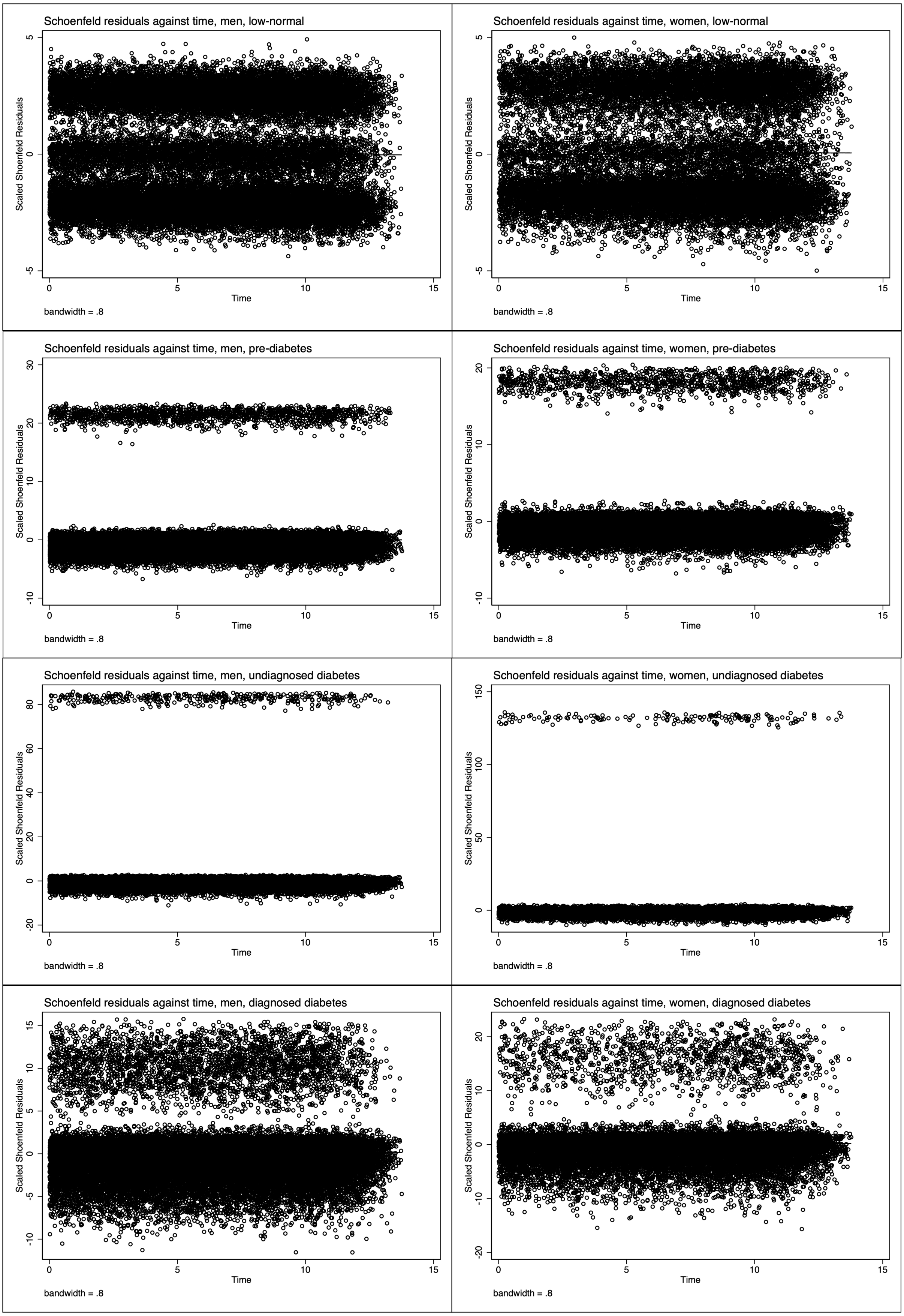

The RECORD statement – checklist of items, extended from the STROBE statement, that should be reported in observational studies using routinely collected health data

	Item No.	STROBE items	Location in manuscript where items are reported	RECORD items	Location in manuscript where items are reported
**Title and abstract**					
	1	(a) Indicate the study's design with a commonly used term in the title or the abstract (b) Provide in the abstract an informative and balanced summary of what was done and what was found	(a) Title & Abstract<br><br>(b) Abstract	RECORD 1.1: The type of data used should be specified in the title or abstract. When possible, the name of the databases used should be included.<br><br>RECORD 1.2: If applicable, the geographic region and timeframe within which the study took place should be reported in the title or abstract.<br><br>RECORD 1.3: If linkage between databases was conducted for the study, this should be clearly stated in the title or abstract.	1.1: Abstract – Methods<br><br>1.2: Abstract – Methods & Findings<br><br>1.3: Abstract – Methods
**Introduction**					
Background rationale	2	Explain the scientific background and rationale for the investigation being reported	Background (Para 1 & 2)		
Objectives	3	State specific objectives, including any prespecified hypotheses	Background (End of Para 3)		
**Methods**					
Study Design	4	Present key elements of study design early in the paper	Methods (Study design and population)		
Setting	5	Describe the setting, locations, and relevant dates, including periods of recruitment, exposure, follow-up, and data collection	Methods (Study design and population; Exposure,		

			outcome and follow-up)		
Participants	6	*(a) Cohort study* - Give the eligibility criteria, and the sources and methods of selection of participants. Describe methods of follow-up<br>*Case-control study* - Give the eligibility criteria, and the sources and methods of case ascertainment and control selection. Give the rationale for the choice of cases and controls<br>*Cross-sectional study* - Give the eligibility criteria, and the sources and methods of selection of participants<br><br>*(b) Cohort study* - For matched studies, give matching criteria and number of exposed and unexposed<br>*Case-control study* - For matched studies, give matching criteria and the number of controls per case	(a) Methods (Study design and population; Exposure, outcome and follow-up)	RECORD 6.1: The methods of study population selection (such as codes or algorithms used to identify subjects) should be listed in detail. If this is not possible, an explanation should be provided.<br><br>RECORD 6.2: Any validation studies of the codes or algorithms used to select the population should be referenced. If validation was conducted for this study and not published elsewhere, detailed methods and results should be provided.<br><br>RECORD 6.3: If the study involved linkage of databases, consider use of a flow diagram or other graphical display to demonstrate the data linkage process, including the number of individuals with linked data at each stage.	6.1: Methods (Study design and population; Exposure, outcome and follow-up)<br><br>6.2: Methods (Exposure, outcome and follow-up; Covariates) & Supplementary Appendix<br><br>6.3: Figure 1
Variables	7	Clearly define all outcomes, exposures, predictors, potential confounders, and effect modifiers. Give diagnostic criteria, if applicable.	Methods (Exposure, outcome and follow-up; Covariates)	RECORD 7.1: A complete list of codes and algorithms used to classify exposures, outcomes, confounders, and effect modifiers should be provided. If these cannot be reported, an explanation should be provided.	7.1: Methods (Exposure, outcome and follow-up; Covariates) & Supplementary Appendix
Data sources/ measurement	8	For each variable of interest, give sources of data and details of methods of assessment (measurement).	Methods (Exposure, outcome and follow-up; Covariates).		

		Describe comparability of assessment methods if there is more than one group			
Bias	9	Describe any efforts to address potential sources of bias	Methods (Covariates; Statistical analysis; Sensitivity analyses)		
Study size	10	Explain how the study size was arrived at	Methods (all)		
Quantitative variables	11	Explain how quantitative variables were handled in the analyses. If applicable, describe which groupings were chosen, and why	Methods (Exposure, outcome and follow-up; Statistical analysis)		
Statistical methods	12	(a) Describe all statistical methods, including those used to control for confounding<br>(b) Describe any methods used to examine subgroups and interactions<br>(c) Explain how missing data were addressed<br>(d) *Cohort study* - If applicable, explain how loss to follow-up was addressed<br>*Case-control study* - If applicable, explain how matching of cases and controls was addressed<br>*Cross-sectional study* - If applicable, describe analytical methods taking account of sampling strategy<br>(e) Describe any sensitivity analyses	(a-c) Methods (Covariates; Statistical analysis; Sensitivity analyses)<br><br>(d) Not applicable for this study<br><br>(e) Methods (Sensitivity analyses)		
Data access and cleaning methods		..		RECORD 12.1: Authors should describe the extent to which the investigators had access to the database population used to create the study population.	12.1: Methods (Role of the funding source)<br><br>12.2: Methods (Study design and

				RECORD 12.2: Authors should provide information on the data cleaning methods used in the study.	population; Covariates)
Linkage		..		RECORD 12.3: State whether the study included person-level, institutional-level, or other data linkage across two or more databases. The methods of linkage and methods of linkage quality evaluation should be provided.	Methods (Study design and population)
**Results**					
Participants	13	(a) Report the numbers of individuals at each stage of the study (*e.g.*, numbers potentially eligible, examined for eligibility, confirmed eligible, included in the study, completing follow-up, and analysed)<br>(b) Give reasons for non-participation at each stage.<br>(c) Consider use of a flow diagram	(a-c) Results (Cohort description; Figure 1)	RECORD 13.1: Describe in detail the selection of the persons included in the study (*i.e.,* study population selection) including filtering based on data quality, data availability and linkage. The selection of included persons can be described in the text and/or by means of the study flow diagram.	13.1: Results (Cohort description; Figure 1)
Descriptive data	14	(a) Give characteristics of study participants (*e.g.*, demographic, clinical, social) and information on exposures and potential confounders<br>(b) Indicate the number of participants with missing data for each variable of interest<br>(c) *Cohort study* - summarise follow-up time (*e.g.*, average and total amount)	(a) Results (Cohort description; Table 1)<br><br>(b) Methods (Covariates); Table 1<br><br>(c) Results (Age-standardised incidence rates)		
Outcome data	15	*Cohort study* - Report numbers of outcome events or summary measures over time<br>*Case-control study* - Report numbers in each exposure category, or summary measures of exposure	Results (Age-standardised incidence rates)		

		Cross-sectional study - Report numbers of outcome events or summary measures			
Main results	16	(a) Give unadjusted estimates and, if applicable, confounder-adjusted estimates and their precision (e.g., 95% confidence interval). Make clear which confounders were adjusted for and why they were included<br>(b) Report category boundaries when continuous variables were categorized<br>(c) If relevant, consider translating estimates of relative risk into absolute risk for a meaningful time period	(a) Results (Figure 2; eTable 2)<br><br>(b) Results (all)<br><br>(c) Results (Age-standardised incidence rates)		
Other analyses	17	Report other analyses done—e.g., analyses of subgroups and interactions, and sensitivity analyses	Results (HbA1c category and CVD risk; Identifying factors most responsible for attenuating excess risk; Sensitivity analyses)		
**Discussion**					
Key results	18	Summarise key results with reference to study objectives	Discussion (para 1)		
Limitations	19	Discuss limitations of the study, taking into account sources of potential bias or imprecision. Discuss both direction and magnitude of any potential bias	Discussion (para 6)	RECORD 19.1: Discuss the implications of using data that were not created or collected to answer the specific research question(s). Include discussion of misclassification bias, unmeasured confounding, missing data, and changing eligibility over time, as they pertain to the study being reported.	19.1 Discussion (para 6)
Interpretation	20	Give a cautious overall interpretation of results considering objectives, limitations, multiplicity of	Discussion (Throughout)		

		analyses, results from similar studies, and other relevant evidence			
Generalisability	21	Discuss the generalisability (external validity) of the study results	Discussion (para 6)		
**Other Information**					
Funding	22	Give the source of funding and the role of the funders for the present study and, if applicable, for the original study on which the present article is based	Methods (Role of the funding source); Funding statement		
Accessibility of protocol, raw data, and programming code		..		RECORD 22.1: Authors should provide information on how to access any supplemental information such as the study protocol, raw data, or programming code.	Data availability statement
